# Septin 9 induces lipid droplets growth by a phosphatidylinositol-5-phosphate and microtubule-dependent mechanism hijacked by HCV

**DOI:** 10.1038/ncomms12203

**Published:** 2016-07-15

**Authors:** Abdellah Akil, Juan Peng, Mohyeddine Omrane, Claire Gondeau, Christophe Desterke, Mickaël Marin, Hélène Tronchère, Cyntia Taveneau, Sokhavuth Sar, Philippe Briolotti, Soumaya Benjelloun, Abdelaziz Benjouad, Patrick Maurel, Valérie Thiers, Stéphane Bressanelli, Didier Samuel, Christian Bréchot, Ama Gassama-Diagne

**Affiliations:** 1 INSERM, Unité 1193, F-94800 Villejuif, France; 2 University of Paris-Sud, UMR-S 1193, F-94800 Villejuif, France; 3 Laboratoire des Hépatites Virales, Département de Virologie. Institut Pasteur du Maroc, BP 20360 Casablanca, Maroc; 4 Faculté des Sciences, Laboratoire de Biochimie-Immunologie, Univ. Mohammed V, Rabat, Maroc; 5 DHU Hepatinov, Villejuif F-94800, France; 6 INSERM U1183, Institute of Regenerative Medicine and Biotherapy, University of Montpellier, 34295 Montpellier, France; 7 Department of Hepato-Gastroenterology A, Hospital Saint Eloi, CHRU, 34295 Montpellier, France; 8 University of Paris-Sud, -UFR medecine- INSERM UMS33, Villejuif, France; 9 INSERM U1048, I2MC and Université Paul Sabatier, 31432 Toulouse, France; 10 Virologie Moléculaire et Structurale CNRS UPR 3296 - INRA UsC 1358, 91198 Gif-sur-Yvette, France; 11 Univ. Internationale de Rabat, Sala Al Jadida, Maroc; 12 Institut Pasteur, 75724 Paris, France; 13 AP-HP Hôpital Paul-Brousse, Centre Hépato-Biliaire, Villejuif F-94800, France

## Abstract

The accumulation of lipid droplets (LD) is frequently observed in hepatitis C virus (HCV) infection and represents an important risk factor for the development of liver steatosis and cirrhosis. The mechanisms of LD biogenesis and growth remain open questions. Here, transcriptome analysis reveals a significant upregulation of septin 9 in HCV-induced cirrhosis compared with the normal liver. HCV infection increases septin 9 expression and induces its assembly into filaments. Septin 9 regulates LD growth and perinuclear accumulation in a manner dependent on dynamic microtubules. The effects of septin 9 on LDs are also dependent on binding to PtdIns5P, which, in turn, controls the formation of septin 9 filaments and its interaction with microtubules. This previously undescribed cooperation between PtdIns5P and septin 9 regulates oleate-induced accumulation of LDs. Overall, our data offer a novel route for LD growth through the involvement of a septin 9/PtdIns5P signalling pathway.

## Abstract (web summary)

The accumulation of lipid droplets is often observed in hepatitis C virus infection, but the mechanism of their formation is not known. Here the authors show that septin 9 expression is increased in infected livers, and a septin 9/phosphatidylinositol 5-phosphate signalling pathway regulates the growth of lipid droplets.

## Graphical abstract

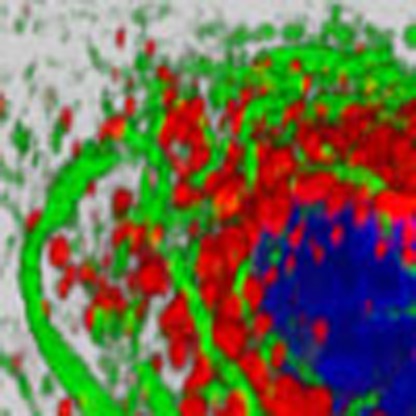

Lipid droplets (LDs) are highly dynamic organelles generated from the endoplasmic reticulum (ER)^1^, which can remain attached to the ER or generate contacts with other organelles, including the mitochondria, lysosomes, peroxisomes and Golgi^2^. LDs consist of a hydrophobic core of neutral lipids, which are primarily composed of triacylglycerols (TAGs) and cholesterol esters, surrounded by a monolayer of phospholipids and a large group of proteins that facilitate cellular signalling interactions, control the access of metabolic enzymes, and influence LD movement within the cell^1^,^3^. These proteins include predominantly the perilipin family (Plins) and proteins of the rab family of GTPases^1^,^4^. There are five known perilipins (PLINs 1–5) expressed in hepatocytes, which play distinct roles. PLIN2 also called ADRP (adipose differentiation-related protein) is a prominent LD protein, expression level of which is tied to LD accumulation. PLIN3 initially described as a tail-interacting protein of 47 kDa (TIP47) is also ubiquitously expressed^5^. LD growth and degradation are highly regulated processes and excessive accumulation of LDs in the liver causes steatosis, which may contributes to cirrhosis, the final stage for several chronic liver diseases and often evolves to hepatocellular carcinoma.

Chronic HCV infection is a major public health problem and a main risk factor for cirrhosis, and hepatocellular carcinoma, thus remaining the major cause of liver resection and transplantation^6^. There is no vaccine to protect against HCV, although major advances have been recently achieved regarding HCV infection treatment, and the combination of drugs, including protease inhibitors, represents a real breakthrough that cure almost 90% of infected patients. However, these treatments have remained very costly. Additionally, understanding the key mechanisms driving HCV replication and persistence will have a profound impact, beyond HCV, for the appraisal of the pathogenesis of this family of viruses. Each step of the replication cycle of HCV is tightly dependent on the host lipid metabolism, and liver steatosis associated with the accumulation of LDs is a hallmark of chronic HCV infection^7^,^8^. HCV and LD interactions are crucial for persistent viral propagation and virion production^9^.

HCV is an enveloped positive single-stranded RNA virus from the Flaviviridae family. HCV genomic RNA encodes for a polyprotein precursor of ∼3,100 amino acids post-translationally cleaved by cellular and viral proteases into the structural (core, E1 and E2), non-structural (NS) proteins (NS2, NS3, NS4A, NS4B, NS5A and NS5B) and p7 protein^8^. The core protein plays a major role in the virion assembly through interaction with LDs^10^. The proteins covering the LD surface and their link with HCV life cycle have been characterized, whereas the potential role of phospholipids in these events remains unclear. The composition of the phospholipid monolayer of LDs depends on the cell type, but is mostly composed of phosphatidylcholine, phosphatidylethanolamine, and, to a lesser extent, phosphatidylinositol (PtdIns) and lysophospholipids^1^,^11^,^12^. The phosphorylated derivatives of PtdIns (PIs) are important signalling molecules that are essential for a variety of cellular functions, including membrane remodelling and trafficking^13^. Although recent studies revealed the role of PtdIns 4-kinases (PI4K-IIIα and PI4K-IIIβ) and their lipid products, PtdIns4P, as critical regulators of the HCV life cycle^8^. The role of class II phosphoinositide 3-kinase (PI3K) was also demonstrated^14^. Furthermore we demonstrated that PIs control epithelial morphogenesis and polarity^15^,^16^ and represent HCV cellular targets^17^.

Septins form a family of GTP-binding proteins composed of 13 members in mammals and conserved from yeast to humans^18^. The complexity of this gene family is increased by the existence of alternative splicing, which markedly increases the number of potential isoforms. Septins assemble into complexes variable in size and composition between organisms^19^,^20^. In drosophila and mammalian septins, complexes contain in general three to four different septins present in two copies. The core hetero-oligomeric units of six to eight subunits serve to build higher order structures, including filaments and rings structures, which are important for septin *in vivo* biological function^20^. Septins are implicated in multiple cellular functions, including cytokinesis, ciliogenesis, cell migration, vesicle trafficking, and cell polarity^21^,^22^,^23^. Septins associate with the cytoskeleton and are known as a fourth type of cytoskeletal structure^23^. Septins also bind to membranes, specifically to PIs, providing membrane stability and serving as diffusion barriers for membrane proteins^20^,^21^,^23^. Nevertheless, these septin/PI interactions have been scarcely investigated^24^,^25^,^26^ and their link with HCV life cycle and their potential role in LD biogenesis and growth remain unclear. Septins are also involved in infection by pathogens, including bacteria, fungi^23^,^27^,^28^ and HCV virus^29^. Alteration in expression profile of septins are reported in different cancers and we determined that septin 9 is specifically upregulated in liver cancer^30^.

Despite being involved in carcinogenesis, septin 9 expression and function have not been investigated in precancerous diseases such as cirrhosis. In this study, we investigate the expression of septin 9 and the differential expression of the other septins in HCV-induced cirrhosis. We also demonstrate that septin 9 regulates LD growth through binding to PtdIns5P in HCV-infected cells. Of interest, we show that this new mechanism is involved in lipid homoeostasis independently of HCV infection.

## Results

### Transcriptomic analysis of septins in human cirrhosis

**Figure 1:**
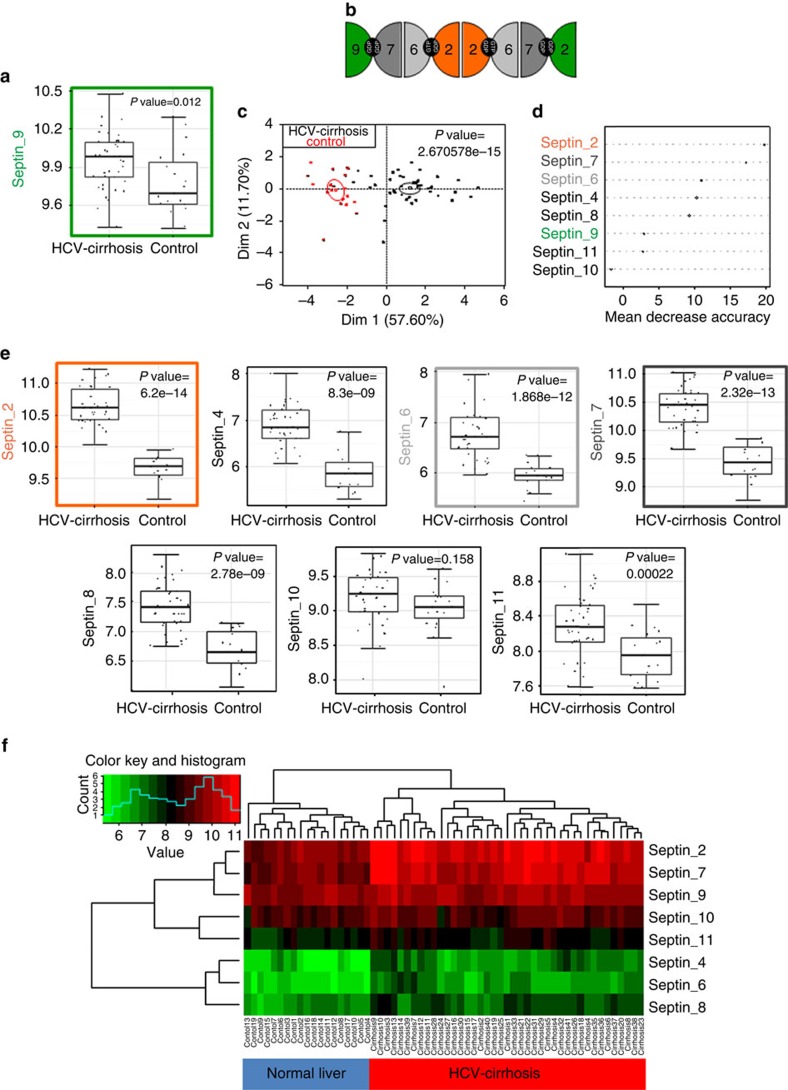
Transcriptomic analysis of septin 9 in human cirrhosis. (**a**) R software was used to generate boxplot presents septin 9 expression in cirrhosis and in normal liver samples of GSE14323 data set and to calculate mentioned *P* value of two-sided Student's *t*-test. (**b**) The octameric complex of septins contains septin 2 group and septin 6 group members, septin 7 and septin 9 which caps the two extremities of the rod shaped complex. (**c**) R software with FactoMineR package was used to obtain unsupervised principal component analysis performed on GSE14323 with septin molecules: *P* value obtained on first principal axis allowed discriminating normal liver from cirrhosis in GSE14323. (**d**) Mean decrease accuracy of predict variables during the ‘Septin' Random Forest learning machine. (**e**) R software was used to generate boxplot of septins allows to discriminate cirrhosis from normal liver with GSE14323 and to calculate mentioned *P* value of two-sided Student's *t*-test. (**f**) R software was used to perform heatmap by unsupervised classification with septin molecules in GSE14323 (classification tree with Euclidean distance and complete linkage).

To determine the expression profile of septin 9 in cirrhosis, we used the GSE14323 data set^31^, including transcriptomes of normal liver (*n*=19) and HCV-induced cirrhosis samples (*n*=49). Data revealed a significant upregulation (two-sided Student's *t*-test, *P*=0.012) of septin 9 transcriptional expression in cirrhosis samples compared to that in normal liver samples (Fig. 1a).

Septin 9 occupies a terminal position in an octameric septin complex involved in the formation of higher order structures such as filaments and rings (Fig. 1b)^32^. According to the importance of hetero-oligomerisation in the assemblies and functions of septins^23^, we assessed the differential expression of septins in HCV-induced cirrhosis using the GSE14323 data set by unsupervised principal component analysis (Fig. 1c–f). Sample projection on the first factorial map showed a significant separation between the normal liver and HCV-induced cirrhosis samples according to the first axis (*P*=2.67E−15) (Fig. 1c). Learning machine algorithm of Random Forest confirmed a good separation of the normal liver and cirrhosis samples. The classification error by class was low and especially null for the cirrhosis class after building 500 classification trees and multidimensional Scaling plot (MDSplot), confirming the good separation of the samples after learning machine analysis (Fig. 1d; Supplementary Fig. 1). Random Forest analysis predicted that most septins are affected in HCV-induced cirrhosis when compared to normal liver, while septin 10 was less affected. This was confirmed by individual boxplot performed on each septin (Fig. 1e, *P* value were calculated by two-sided Student *t*-test). These analyses showed that the expression of septin 7 and 2 is massively affected in these samples (Fig. 1d,e). A weak error on misclassification by class (confusion of 1.67%) was detected when using the learning machine algorithm (Supplementary Fig. 1c). Thus, we performed an unsupervised classification on these samples with previously used septin expression (classification tree with Euclidean distance and complete linkage (Fig. 1f)). This unsupervised algorithm performed on 58 clinical samples allowed the discrimination of two major groups of experiments with no error of misclassification between normal liver and HCV-induced cirrhosis samples. This result suggests that the expression of septin protein family is deregulated in HCV-induced cirrhosis as compared with that in the normal liver.

### HCV assembles septin 9 which stabilizes microtubules

**Figure 2:**
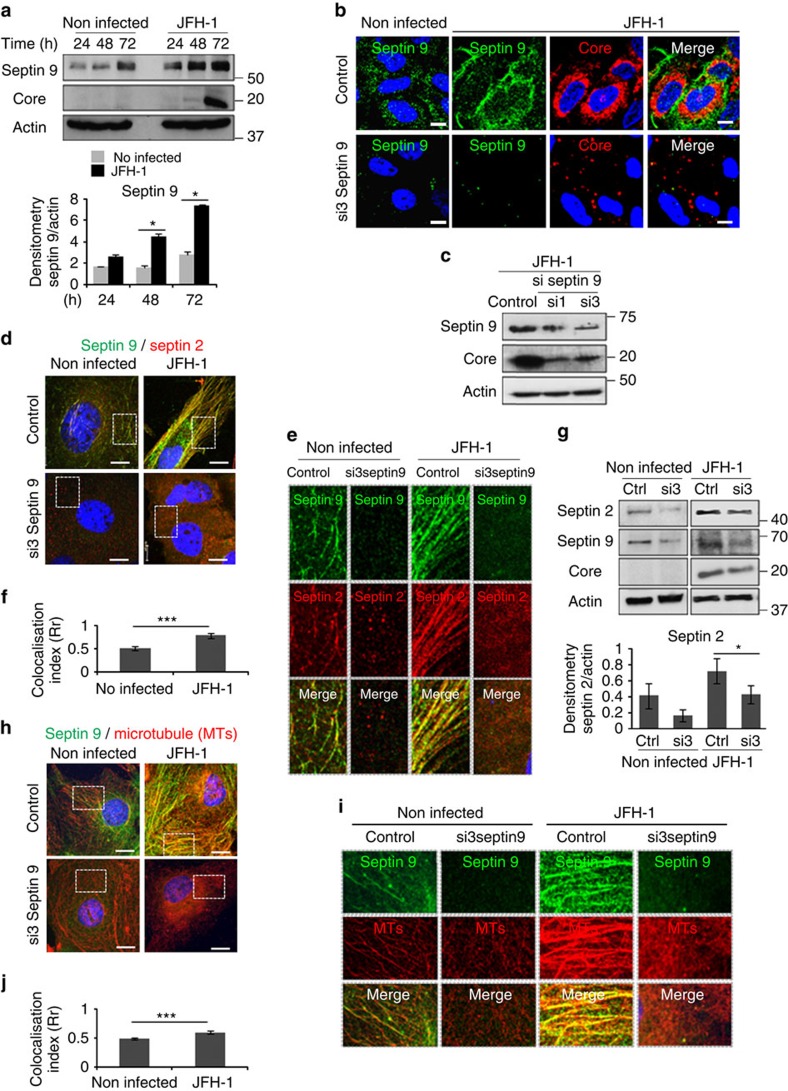
Septin 9 increases in JFH-1-infected cells and regulates septin 2 and microtubules filaments. (**a**) Immunoblot of septin 9 and core in Huh7.5 cells infected or not with JFH-1 for 24, 48 and 72 h. Actin was used as a loading control. The bar graph presents the densitometry analysis of the immunoblots from three independent experiments. (**b**) Huh7.5 cells transfected with non-targeting (control) or septin 9 siRNA (si3) for 24 h then infected or not with HCV JFH-1 for 72 h then stained for septin 9 (green) and core (red). (**c**) Immunoblot of septin 9 and core in Huh7.5 cells transfected with non-targeting (control) or septin 9 siRNA (si1 or si3) then infected with HCV JFH-1 for 72 h. (**d**) Huh7.5 treated as in **b** and stained for septin 9 (green) and septin 2 (red). (**e**) Dot rectangles in **d** are presented in higher magnification. (**f**) Bar graph shows Pearson's correlation coefficient (Rr) of septin 9 and septin 2 calculated in 30 cells from 2 independent experiments. (**g**) Immunoblot of septin 2, septin 9 and core in Huh7.5 cells treated as described in **b**. Actin was used as a loading control. Bar graph presents septin 2 expression from three independent experiments. (**h**) Huh7.5 cells were treated as described in **b** and stained for microtubules (MTs) with β tubulin (red) and septin 9 (green). (**i**) Dot squares in **h** present in higher magnification. (**j**) Bar graph shows Pearson's correlation coefficient (Rr) analysis of septin 9 and MTs calculated in 30 cells from two independent experiments. Values are means±s.e.m. Student's *t*-test was used. *P<0.05, ***P<0.0001. Scale bar, 10 μm.

Following the above described transcriptomic study, we hypothesized that HCV could be responsible of the upregulation of septin 9 in cirrhosis. To explore this possibility, Huh7.5 cells were inoculated for 24, 48 and 72 h with HCV JFH-1 (Japanese fulminate hepatitis 1), a genotype 2a strain, which produces infectious particles in culture and recapitulates all steps of the HCV life cycle^33^. Immunoblot data revealed a non-significant, time-dependent increase of septin 9 expression in non-infected cells, while septin 9 expression was significantly enhanced in JFH-1-infected cells (Fig. 2a), with the highest expression at 72 h of infection (Fig. 2a) when septin 9 formed bundles of filaments surrounding the clusters of HCV core (Fig. 2b). Treatment of Huh7.5 cells with two different septin 9 siRNAs (si1 and si3) before their infection strongly reduced core expression (Fig. 2b,c). These data suggested that HCV-regulated septin 9 assembly and expression was required for core protein expression.

Thus, to further characterize the septin 9 filamentous structure, we determined the presence of other septins. We focussed on septin 2, the major ubiquitously expressed septin and the most deregulated in HCV-induced cirrhosis (Fig. 1). In non-infected cells, septin 2 formed short filaments that co-localized with septin 9 (Fig. 2d,e). These filamentous structures containing septin 2 and their co-localization with septin 9 were significantly increased upon JFH-1 infection (Fig. 2d–f). Depletion of septin 9 using si3 disrupted septin 2 filaments and reduced its staining when compared with that in control cells in both non-infected and JFH-1-infected cells (Fig. 2d,e). These data were confirmed by immunoblot analysis (Fig. 2g) and suggested the role of septin 9 in the formation of septin filamentous structure, consistent with previous reports^32^.

Next, we assessed the effects of septin 9 expression on HCV-modulated microtubule (MT) network. Tubulin staining indicated a strong MT network in HCV-infected cells when compared with that of non-infected cells (Fig. 2h,i; Supplementary Fig. 2a). Importantly, MTs co-localized with septin 9 filaments and si3 severely destroyed MT filaments (Fig. 2h–j), indicating a regulatory role of septin 9 in HCV-dependent MT organization.

### Septin 9 regulates LD behaviour and HCV replication

**Figure 3:**
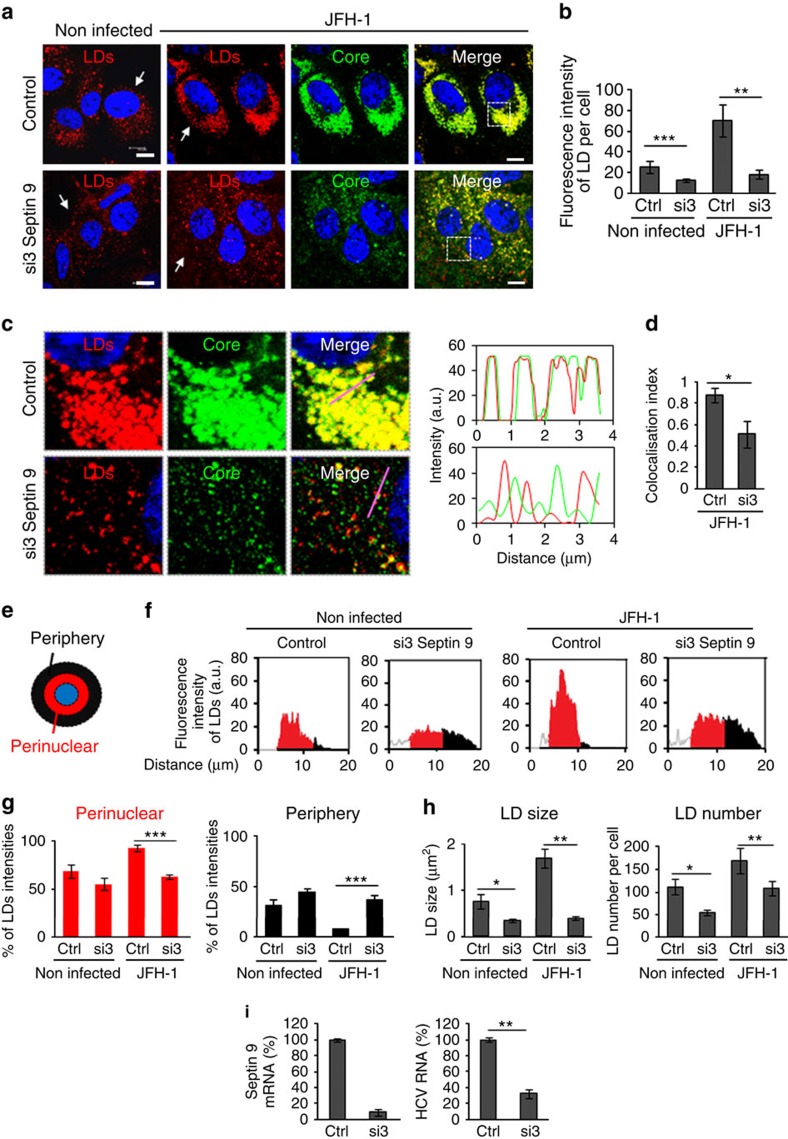
Septin 9 regulates core and LDs accumulation in JFH-1 infected cells and virus replication. (**a**) Huh7.5 cells were transfected with non-targeting (control or Ctrl) or septin 9 siRNA (si3) for 24 h then infected with JFH-1 for further 72 h and stained for core (green) and LDs (red). (**b**) LDs fluorescence intensity in 30 cells from two experiments performed as described in **a**. (**c**) Dot squares in **a** are presented in higher magnification. The right panels present green, red, line profile blots of the pink lines. (**d**) Bar graphs show Pearson's correlation coefficient (Rr) for co-localization between core and LDs of 30 cells from two experiments. (**e**) Representation of the peripheral (black) and perinuclear (red) regions of the cell. (**f**) Radial profile plots showing LD distribution between the peripheral (black) and the perinuclear regions (red) of cells indicated with the white arrows in **a**. (**g**) Quantification of LDs in perinuclear and peripheral regions in 30 cells from two independent experiments performed as described in **a**. (**h**) LD size (left) and LD number (right) in 30 cells from two independent experiments done as described in **a**. (**i**) Huh7.5 cells were transfected with non-targeting (control) or septin 9 siRNA (si3) for 24 h and infected with JFH-1 as in **a** for 72 h. Cells were analysed for septin 9 mRNA and HCV RNA level by qRT–PCR. Bar graphs show results from 3 independent experiments. Values are means±s.e.m. Student's *t*-test was used. *P<0.05, **P<0.001, ***P<0.0001. Scale bar, 10 μm.

**Figure 4:**
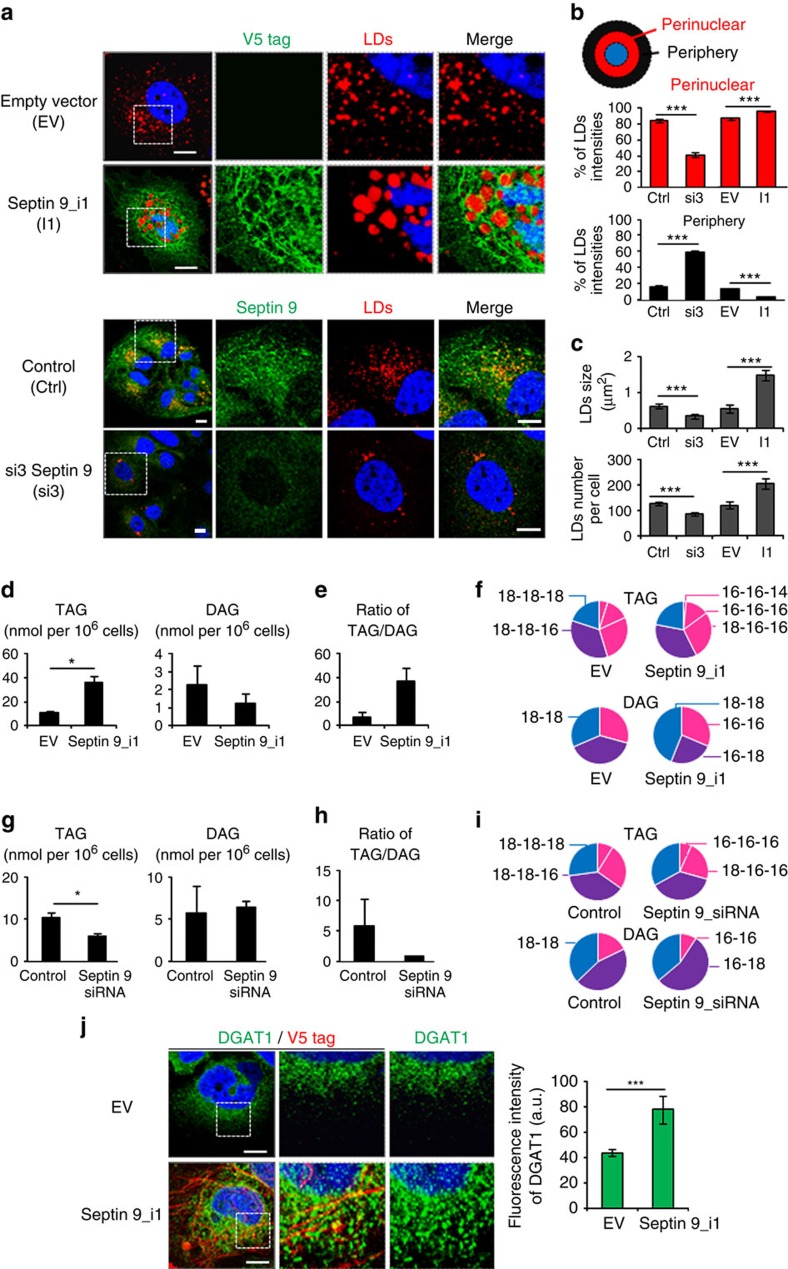
Septin 9 regulates TAG and DAG levels in Huh7R cells. (**a**) Huh7R cells transfected with empty vector (EV) or septin 9_i1 for 48 h were stained for LDs (red) and V5 tag (green). Cells transfected with non-targeting (control) or septin 9 siRNA (si3) for 48 h were stained for LDs (red) and septin 9 (green). (**b**) Schematic representation shows the perinuclear and peripheral regions of the cell. Bar graphs represent LDs intensity in the perinuclear and peripheral region of 36 cells from five independent experiments. (**c**) LD size and LD number in 36 cells from 5 independent experiments. (**d**–**f**) Huh7R cells were transfected with either EV or septin 9_i1 cDNAs then analysed for triacylglycerol (TAG) and diacylglycerol (DAG). (**d**) Data represent nmol per 10^6^ cells. (**e**) Ratio of TAG to DAG. (**f**) Distribution of fatty acid species in TAG and DAG according to carbon number in the acyl group. Results are obtained from 3 independents experiments. (**g**–**i**) Huh7R cells transfected with non-targeting or septin 9 siRNA for 72 h were analysed for TAG and DAG as in **d**–**f**. (**j**) Huh7R cells transfected with EV or septin 9_i1 were stained for V5 tag (red) and diacylglycerol acyltransferase-1 (DGAT1) (green). White squares indicate the area shown in higher magnification. Bar graph represents DGAT1 fluorescence intensity in 50 cells from two independent experiments. Values are means±s.e.m. Student's *t*-test was used. *P<0.05, ***P<0.0001. Scale bar, 10 μm.

The accumulation of LDs is an important feature of HCV infection^4^. Thus, to understate the importance of septin 9 in HCV life cycle, we investigated LDs. JFH-1 infection sharply increased LDs that formed clusters co-localized with the core protein around the nucleus (Fig. 3a), while si3 treatment markedly decreased LDs (Fig. 3b) and their co-localization with core (Fig. 3c,d). A typical profile of LD distribution was presented (Fig. 3e,f). In non-infected cells, about 70% of LDs were present in the perinuclear area and si3 treatment decreased the percentage to 55%. JFH-1 infection increased the perinuclear LDs up to 92% and septin 9 depletion decreased this level to 62% (Fig. 3g). These changes in the level and distribution of LDs were accompanied by a 2.5-fold increase of LD size in JFH-1-infected cells, while si3 decreased the LD size by half (Fig. 3h). Similar changes were observed for LD number (Fig. 3h). PLIN2 is among the best studied protein associated with LDs and is considered as a marker of LDs^34^. The strong increase in PLIN2 expression and its co-localization with core observed in JFH-1-infected cells were abolished by si3 treatment (Supplementary Fig. 3) and emphasized the role of septin 9 in HCV-induced LD accumulation. Finally, we revealed a strong decrease of HCV genomic RNA after septin 9 knockdown (Fig. 3i), suggesting a critical role of septin 9 in HCV replication. We also analysed the transcript level of the five well-characterized variants of septin 9 (i1 to 5) after JFH-1 infection. Isoform 1 mRNAs were the most abundant, while isoform 3 mRNAs were almost undetectable (Supplementary Fig. 2b). Accordingly, the following studies focused on septin 9 isoform 1 (Septin 9_i1) and we used Huh7 cells stably expressing HCV full-length genomic replicon from genotype 1b (Huh7R)^35^. This system permitted the efficient expression of all viral proteins and is a convenient and valuable tool for molecular studies. A decrease in both the core and envelope E2 proteins was observed in Huh7R cells transfected with si3 (Supplementary Fig. 4a). By opposite both core and E2 expression increased in cells expressing septin 9_i1 compared with control cells (Supplementary Fig. 4b). Later, septin 9_i1 and the LD characteristics were assessed by immunofluorescence analysis. Septin 9_i1 assembled in filaments surrounding the large clusters of LD and transfection of si3 inhibited LD accumulation (Fig. 4a). Overall, profiles of LD distribution and changes in LD morphology (Fig. 4b,c) were similar to the results obtained using the JFH-1 strain (Fig. 3). Septin 9_i1 filaments co-localized with endogenous septin 2 (Supplementary Fig. 5a), indicating that septin 9_i1 is integrated in a hero-oligomeric structure composed of other septins consistent with data in Fig. 2. Moreover, the ratio of the intensity of exogenous septin 9_i1 revealed with an anti-V5 tag antibody to the intensity of endogenous septin 9 detected with septin 9 antibody was 1.1 (Supplementary Fig. 5b). Thus, we concluded that septin 9_i1 expression was similar to that of endogenous septin 9.

Although the first evidence for HCV interaction with LDs was obtained from a study on the core protein, recent studies indicated that the interaction of the HCV non-structural protein NS5A with PLIN3 is essential for the recruitment of NS5A on LDs to promote the release of HCV particles^36^,^37^. Interestingly, increases in PLIN3 and NS5A expression were observed in Huh7R cells expressing septin 9_i1 (Supplementary Fig. 6a). These results were confirmed by immunoblot (Supplementary Fig. 6b) indicating that septin 9_i1 regulated LDs growth and HCV protein expression.

### Septin 9 regulates neutral lipid metabolism

LD growth can be mediated by several mechanisms, but, typically, its size expansion involved an increase in the neutral lipid content, which essentially consists of TAG and cholesterol ester^5^. Therefore, we analysed the neutral lipid content of septin 9_i1-expressing cells. Transfection of septin 9_i1 in Huh7R cells induced a threefold increase of TAG and correlated with a decrease of diacylglycerol (DAGs) (Fig. 4d). As a consequence, the ratio of TAG to DAG was greatly increased (Fig. 4e). The fatty acid composition of TAG and DAG was also analysed. The composition of these two lipids was very similar and consisted mostly of C16 and C18 long chains (Fig. 4f). Similar data were obtained using septin 9-depleted Huh7R cells (Fig. 4g-i). A significant TAG decrease and an increase in DAG were observed (Fig. 4g), suggesting a conversion of the TAG pool into DAGs.

Diacylglycerol acyltransferase-1 (DGAT1) is one of the two known DGAT enzymes that catalyse the final step in triglyceride biosynthesis^38^. The role of DGAT1 in the trafficking of the HCV core protein to LDs and its involvement in the production of infectious HCV particles were reported^39^,^40^. Thus, we analysed DGAT expression and we observed a significant increase in the total intensity of the DGAT1 signal in septin 9_i1-expressing cells. Moreover, DGAT1 co-localized with septin 9_i1 filaments, as shown at the highest magnification (Fig. 4j). Together, these data indicated that septin 9 could also participate in LD growth by regulating lipid biosynthesis.

### MTs are required for septin 9-induced LD behaviour

**Figure 5:**
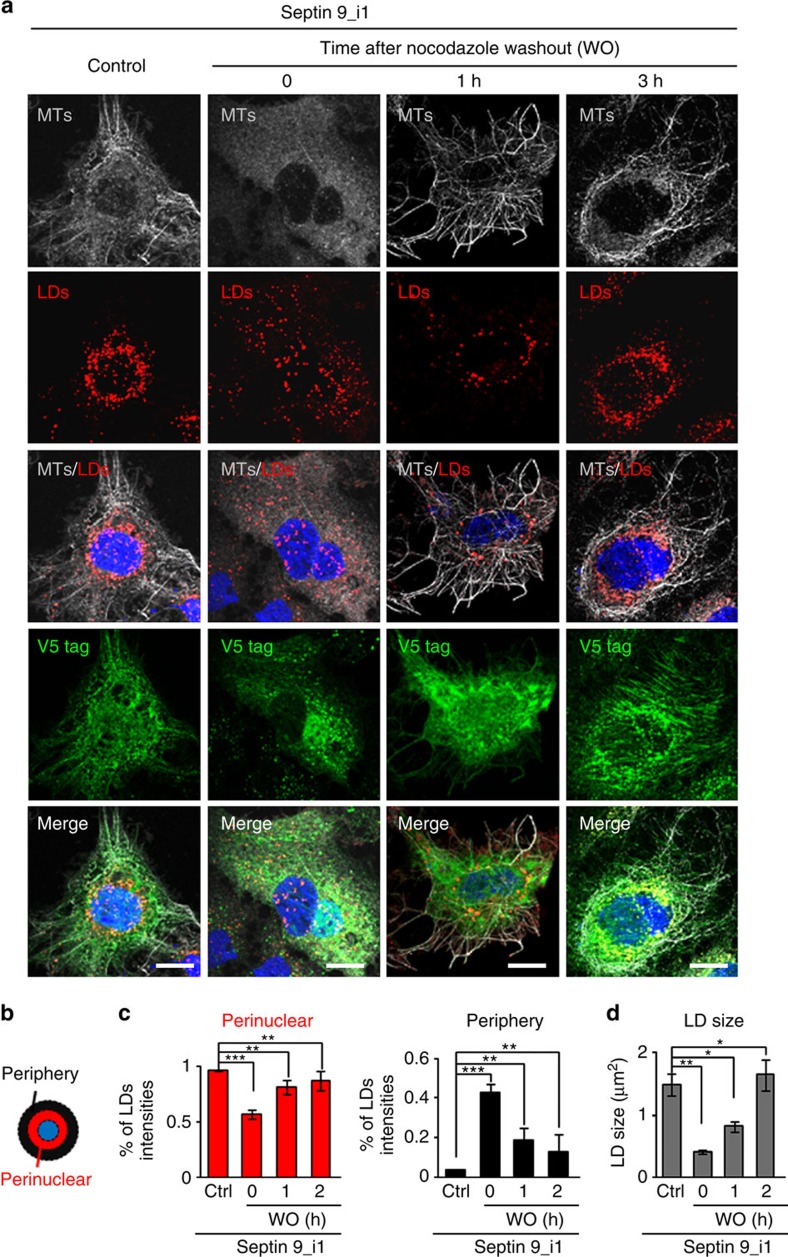
Septin 9 regulates LDs in microtubules-dependent manner. (**a**) Huh7R cells transfected with septin 9_i1 were treated with nocodazole (33 μM) for 2 h at 37 °C and placed on ice for 1 h. Then cells were washed five times with ice-cold culture medium to remove the nocodazole and moved at 37 °C for indicated time in the figure before staining for V5 tag (green), LDs (red) and microtubules (grey) with β tubulin. (**b**) Schematic representation shows the perinuclear and peripheral regions. (**c**) Percentage of LDs intensity in the perinuclear and peripheral regions of 20 cells from two independent experiments. (**d**) LD size analysed in 20 cells from two independent experiments. Values are means±s.e.m. Student's *t*-test was used. *P<0.05, **P<0.001, ***P<0.0001. Scale bar, 10 μm.

Different reports indicated that MTs play a role in the dynamics and growth of LDs^41^,^42^. Furthermore, septins interact with MTs and are essential for MT-dependent cell processes^12^,^23^,^43^. Therefore, we postulated that MTs could play a role in septin 9-induced LD growth. Consequently, we performed a nocodazole washout (Fig. 5). Treatment of Huh7R cells expressing septin 9_i1 with nocodazole resulted in the dispersion of LDs throughout the cell and decreased their size. One hour after nocodazole removal, LDs started to gather around the nucleus and increased in size. These changes were accompanied by the partial recovery of the microtubule filamentous structure (Fig. 5a). Finally, LDs were restored to their initial size and relocated around the nucleus 3 h after nocodazole removal (Fig. 5b–d). Taken together, these results indicate that MT dynamics are required for septin 9 to induce LD growth and perinuclear clustering.

### PtdIns5P binds to septin 9 and promotes LD growth

**Figure 6:**
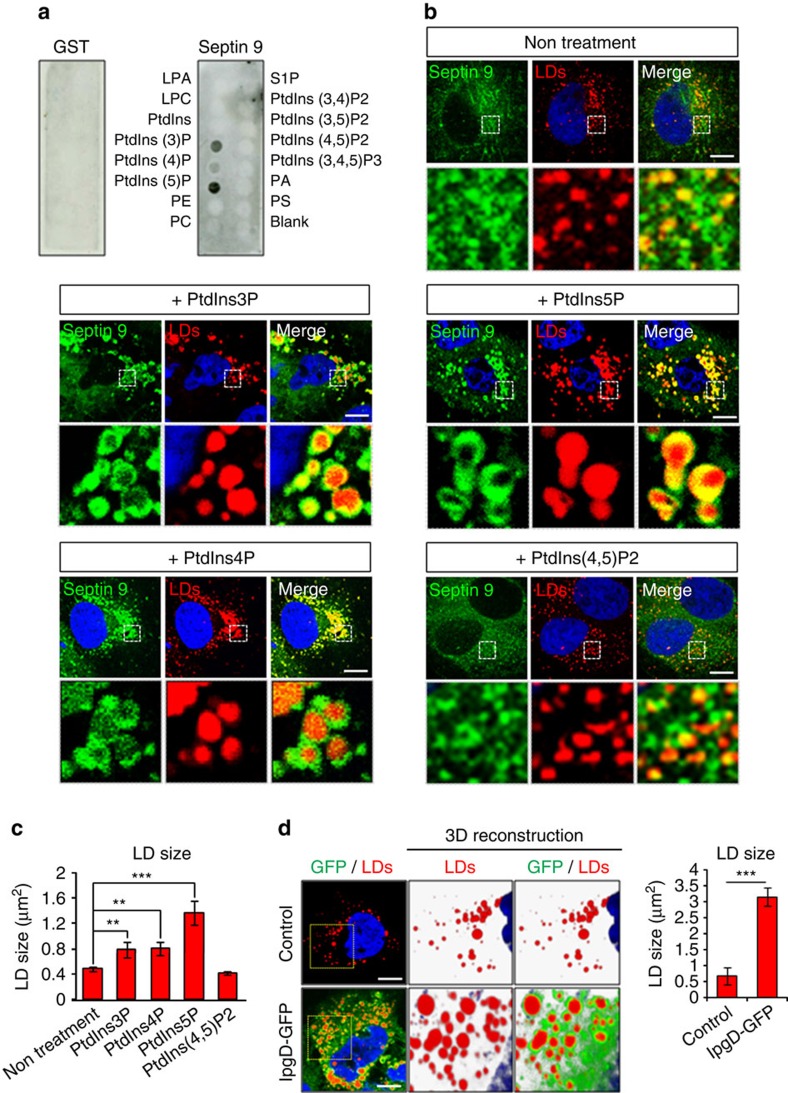
Mono-phosphate phosphoinositides regulate LDs size. (**a**) PIP strip overlay assay: PIP strips were incubated either with glutathion-S-transferase (GST) used as a negative control or with purified septin 9_i3 GST tagged at 0.5 μg ml^−1^ and analysed with anti GST antibody. LPA, lysophosphatidic acid, LPC, lysophosphocholine, PtdIns, phosphatidylinositol, PtdIns(3)P, PtdIns(4)P, PtdIns(5)P, PtdIns(3,4)P2, PtdIns(3,5)P2, PtdIns(4,5)P2, PtdIns(3,4,5)P3, PA, phosphatidic acid, PS, phosphatidylserine, PE, phosphatidylethanolamine, PC, phosphatidylcholine, S1P, sphingosine 1-phosphate. (**b**) Huh7R cells were treated with cell-permeant PtdIns3P, PtdIns4P, PtdIns5P or PtdIns (4, 5) P2 at 30 μM for 15 min before fixing and staining for septin 9 (green) and LDs (red). The dot squares indicate the zoom area. (**c**) Bar graph shows the LD siz in Huh7R cells treated as described in **b**. The results were obtained from at least 20 cells for each treatment from three independent experiments. (**d**) Huh7R cells were transfected with IpgD-GFP construct or not (control) for 48 h and stained for LDs (red). Bar graph represents LDs size measured in 15 cells from two independent experiments. Values are means±s.e.m. Student's *t*-test was used. **P<0.001, ***P<0.0001. Scale bar, 10 μm.

To further identify the mechanism underlying septin 9 assembly and its role on LD, we assessed the importance of phosphoinositides (PI). The phospholipid monolayer that surrounds LDs plays a role in the regulation of LDs morphology. Phosphatidylcholine stabilizes the tension and reduces LD growth, while phosphatidic acid contributes to LD expansion^44^. PtdIns was also detected on LD surface^11^, however, the presence and the role of its phosphorylated derivatives, PIs, on LD growth have not been investigated. Therefore, we identified the PIs that bind to septin 9 by performing a protein–lipid overlay assay using a glutathione-*S*-transferase (GST)-tagged recombinant protein (Fig. 6a). Data from the assay revealed that septin 9 bound specifically to mono-phosphorylated PtdIns (PtdIns3P, PtdIns4P and PtdIns5P) and the strongest interaction was observed with PtdIns5P while no signal was detected using GST protein alone as a negative control (Fig. 6a), supporting the specificity of the observed binding signals.

Furthermore, we showed that PtdIns3P, PtdIns4P and PtdIns5P promoted a significant increase in LD size compared with non-treated cells, whereas PtdIns(4,5)P2 evaluated as a non-binding septin PI, had no significant effect (Fig. 6b,c). PtdIns5P was the most efficient (Fig. 6b,c), consistent with the results in Fig. 6a. Moreover, septin 9 assembled surrounding the LDs that became more visible upon the addition of PtdIns5P (Fig. 6b). Overall, these data revealed the crucial role of mono-phosphorylated PIs in the expansion of LDs and the organization of septin 9 high-order structures. Thus, we focused on PtdIns5P, which was the most effective on LDs. PtdIns5P specifically binds to the plant homeodomain (PHD) finger found in many chromatin-remodelling proteins, and the PHD domain of the tumour suppressor inhibitor of growth (ING2) was proposed to function as a receptor of PtdIns5P (ref. 45). We used the biotinylated GST-tagged PHD domain of ING2 as a probe to visualize cellular PtdIns5P, as previously described^46^. As expected, compared with untreated cells, PtdIns5P addition strongly increased both the PHD signal and the LD size and confirmed that addition of exogenous PtdIns5P increased its intracellular level (Supplementary Fig. 7a–c). To validate the role of PtdIns5P on LDs, we used another approach based on IpgD, the virulence factor of *Shigella flexneri*, which is a PtdIns(4,5)P2-4-phosphatase responsible for the profound increase of PtdIns5P in *S. flexneri*-infected cells^47^. Transfection of Huh7R cells with GFP-tagged-IpgD cDNA (GFP-IpgD) increased PtdIns5P signal (Supplementary Fig. 7d) and LD size and accumulation (Fig. 6d).

### PIKfyve regulates septin 9 filaments and LD accumulation

**Figure 7:**
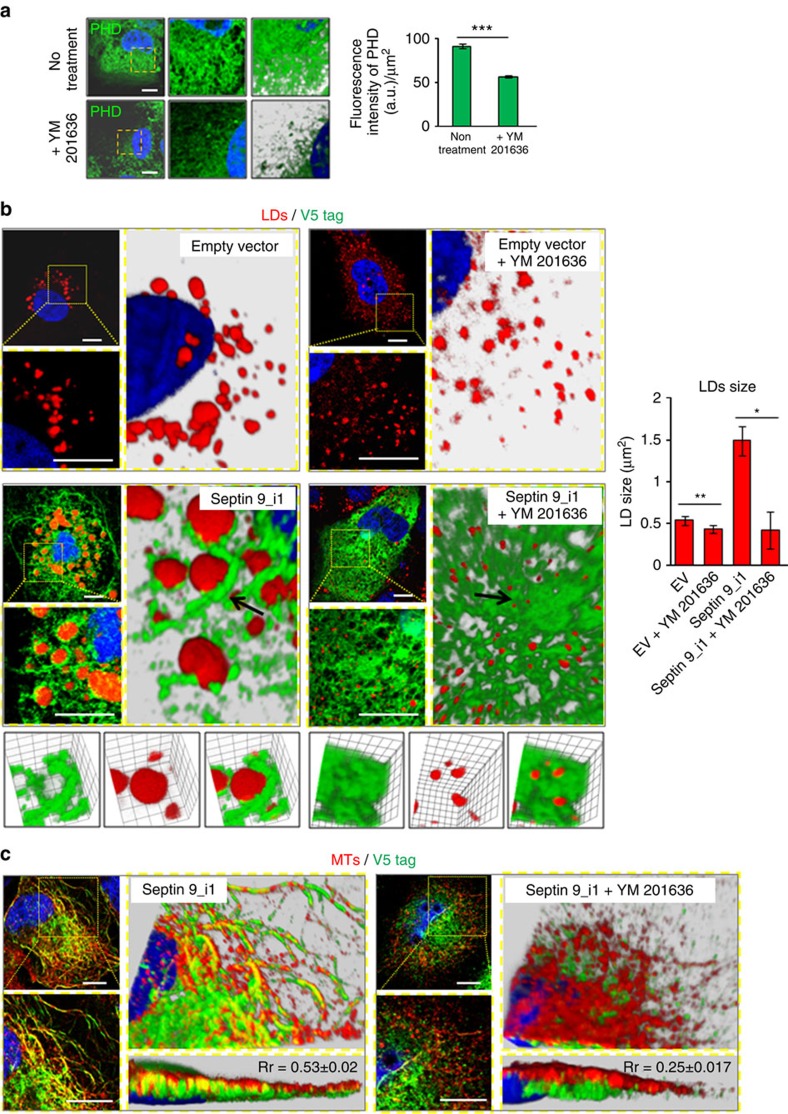
Huh7R treatment with YM201636 causes decrease of LDs size and disrupts septin 9 and microtubules filaments. (**a**) Huh7R cells treated or not with YM201636 were stained for PtdIns5P (PHD) (green). Dot yellow squares indicate the zoomed area shown in 2D image with a black background and in 3D reconstruction images with white background. Bar graph shows PtdIns5P fluorescence intensity analysis of at least in 60 cells from three independent experiments. (**b**) Huh7R cells were transfected with either empty vector (EV) or septin 9_i1 then treated with YM201636 and stained for V5 tag (green) and LDs (red). The dot squares indicate the zoomed area shown in a 2D image with a black background, and to the right is a 3D reconstruction image with a grey background. Arrows indicated the area present below at a higher magnification. Bar graph shows LD size in 30 cells from three experiments. (**c**) Huh7R cells transfected with septin 9_i1 and treated with YM201636 were stained for V5 tag (green) and microtubules (red) with β tubulin. The dot squares indicate the zoomed areas shown as a 2D images with a black background, and to the right is a 3D reconstruction image with a grey background and longitudinal section (right down). Pearson's Correlation coefficient (Rr) for septin and microtubules was calculated from 20 cells from three independent experiments. Values are means±s.e.m. Student's *t*-test was used. *P<0.05, **P<0.001, ***P<0.0001. Scale bar, 10 μm.

To further study the interaction between septin 9 and PtdIns5P and its importance in LD accumulation, we used YM201636, a selective inhibitor of FYVE finger containing phosphoinositide kinase (PIKfyve), a major contributor to the intracellular production of PtdIns5P (ref. 48). First, the effect of YM201636 was verified by PtdIns5P immunostaining in Huh7R cells (Fig. 7a). Subsequently, the cells were transfected either with an empty vector or a septin 9_i1 construct and treated with YM201636. Moreover LD size and level increased in cells expressing septin 9_i1 and were surrounded by septin 9_i1 filaments as described in Fig. 4. YM201636 treatment profoundly reduced the LD size (Fig. 7b,c and Supplementary Fig. 8a) and decreased TAGs concomitantly with an increase of DAGs (Supplementary Fig. 8b–d). Interestingly, YM201636 treatment disrupted septin 9_i1 (Fig. 7b; Supplementary Fig. 8a) and MT filaments and prevented both co-localization (Fig. 7c). Furthermore, PtdIns5P addition to Huh7R cells transfected with septin 9 siRNA partly rescued the disruption of MT filaments and LD clustering caused by septin 9 depletion (Supplementary Fig. 9). Thus, these data demonstrated that PtdIns5P is a crucial regulator of septin 9 structural and functional features.

Interestingly our data revealed that both septin 9 and HCV-regulated PtdIns5P levels. Indeed infection of Huh7.5 cells using JFH-1 particles increased PtdIns5P (Supplementary Fig. 10a). Moreover PtdIns5P increased in Huh7R cells compared with naive Huh7 cells and transfection of the later cells with septin 9_i1 cDNA significantly enhanced the PtdIns5P signal (Supplementary Fig. 10b,c).

### Septin 9 PBR is required for its assembly and LD growth

**Figure 8:**
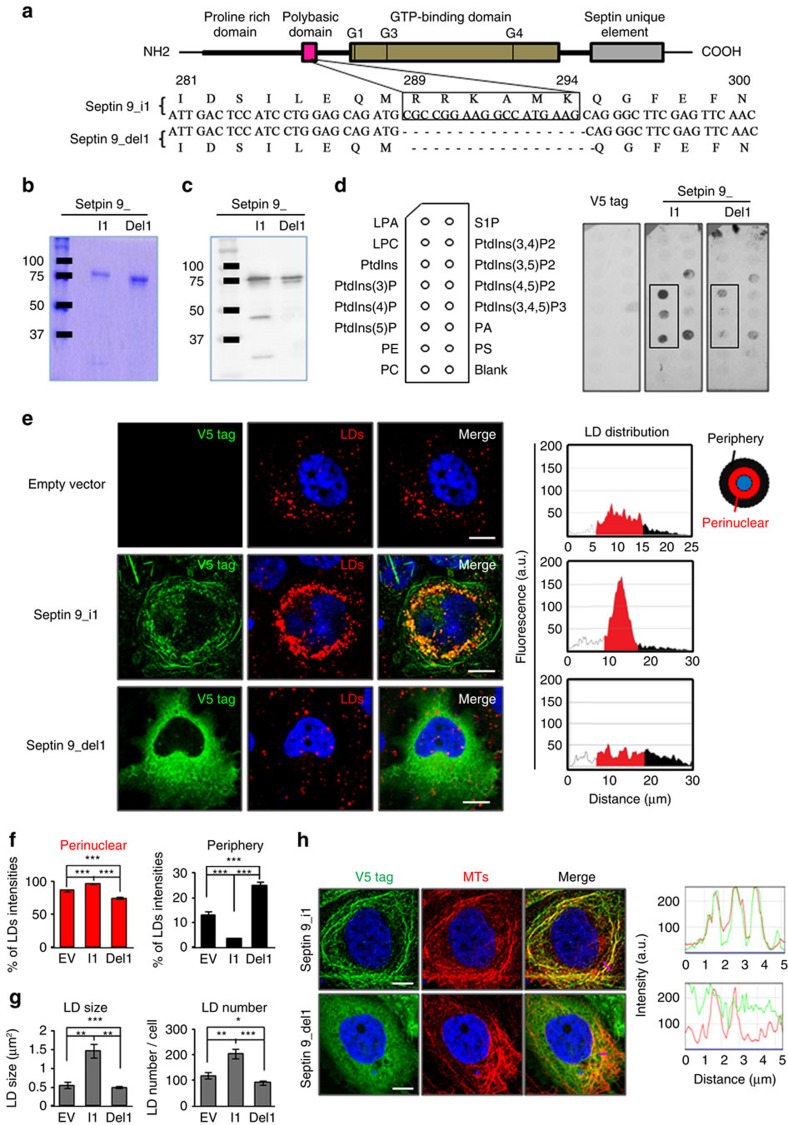
Deletion of septin 9/PIs interaction domain disturbs localization of septin 9 with microtubules and affects LDs accumulation. (**a**) Septin 9 domain organization: the polybasic domain is located at N-terminal of GTP-binding domain, which is recognized by three motifs G1 (GXXXXGK=G^305^QSGLGK^311^), G3 (DXXG=D^362^TPG^365^), G4 (XKXD=A^444^KAD^447^). Down: septin 9_i1 and septin 9_del1 sequence in the polybasic domain region. The boxed sequence (^289^RRKAMK^294^) has been deleted in septin 9_del1. (**b**) Coomassie blue stained SDS–PAGE gel of purified septin 9_i1 and septin 9_del1. (**c**) Immunoblot analysis of purified septin 9_i1 and septin 9_del1. (**d**) PIP strip overlay assay for V5 tag, purified septin 9_i1 and septin 9_del1 performed as in Fig. 6a. Lipid-bound V5 fusion proteins were detected with anti-V5 antibody. (**e**) Left: Huh7R cells were transfected either with EV or septin 9_i1 or septin 9_del1 and stained for V5 tag (green) and LDs (red). Right: radial profile plots of the presented cells show LDs intensity distribution in the peripheral and perinuclear regions. (**f**) LDs intensity in the perinuclear and peripheral regions of 36 cells from 5 independent experiments performed as described in **e**. (**g**) LD size and LD number in 36 cells from 5 independent experiments done as described in **e**. (**h**) Staining of V5 tag (green) and microtubules (MTs) with β tubulin (red) in Huh7R cells transfected with septin 9_i1 or septin 9 del1. The Pearson's correlation coefficient (Rr) measured in 30 cells from two independent experiments is presented on the merge panels. The right panels are green, red, line profile blots of the pink lines. Values are means±s.e.m. Student's *t*-test was used. *P<0.05, **P<0.001, ***P<0.0001. Scale bar, 10 μm.

Septins bind PIs *in vitro* via the polybasic region (PBR) near their N terminus^26^,^49^. The conserved sequence encompassing residues aa289 to aa294 is boxed in Fig. 8a. Thus, as another approach to assess the importance of PI for septin 9 structural and functional features, we generated a PBR-deleted mutant (septin 9_del1) from the cDNA of V5-tagged septin 9_i1. The recombinant proteins from the entire septin 9_i1 and the deleted construct were produced in *Escherichia coli*, purified to near homogeneity, and analysed by SDS gel electrophoresis (Fig. 8b). Coomassie blue staining revealed a major band of the expected molecular weight (70 kDa) for both septin 9_i1 and the deleted mutant, while some lower molecular weight species co-purified with septin 9_i1 (Fig. 8b). Immunoblot (Fig. 8c) confirmed that all detected protein bands were recognized by a V5-tag antibody. Purified recombinant proteins were used to perform a lipid overlay assay. Septin 9_i1 bound PtdIns3P, PtdIns4P, and PtdIns5P as expected, although a signal was observed with phosphatidic acid and PtdIns(3, 5)P2. The lipid-binding signal was strongly reduced for septin 9_del1 (Fig. 8d). Subsequently Huh7R cells^35^ were transfected with the septin 9_i1, septin 9_del1 cDNAs, or empty vector (EV). Septin 9_i1 formed filaments whereas septin 9_del1 could not form filaments (Fig. 8e). Expression of septin 9_i1 increased LD in the perinuclear region compared to EV-transfected cells (Fig. 8e,f). In contrast, septin 9_del1 expression significantly reduced the total level of LDs and their perinuclear distribution (Fig. 8e,f). These changes in the LD distribution were accompanied by significant decrease of the LD size and number (Fig. 8g). Furthermore septin 9_i1 filament structures co-localized with MTs. By contrast, the mutant septin 9_del1 lost the filamentous structure and co-localization with MTs. The Pearson co-localization coefficient (Rr) between septin 9 and MTs dropped from 0.56±0.01 in cells expressing septin 9_i1 to 0.17±0.03 in cells expressing septin 9_del1 and the RGB line profile showed a similarity between MTs and septin 9_i1 cellular distribution (Fig. 8h). These results indicate that PBR is necessary for the formation of septin 9 filamentous structures and LD growth. Moreover, PBR is involved in MTs organization and highlighted the critical role of septin 9 in cytoskeleton organization.

### Septin 9 regulates LD growth in naive cells

Unravelling this new role of septin 9 in LD behaviour led us to examine its relevance independently of HCV infection. We performed a set of experiments using naive Huh7 cells. Septin 9_i1 expression significantly increases LD size comparing to EV-transfected cells (Supplementary Fig. 11a,b). Additionally, neutral lipids analysis revealed an increase TAG in septin 9_i1-transfected cells (Supplementary Fig. 11c,d). Expression of GFP-IpgD (Supplementary Fig. 11e,f) or the addition of exogenous mono-phosphorylated PIs to huh7 cells increase LD size (Supplementary Fig. 12a). In contrast, septin 9_del1 transfection or treatment with YM201636 in septin 9_i1-transfected cells significantly decrease LD size (Supplementary Fig. 12b), disrupted septin 9 filamentous structure and its co-localization with MTs (Supplementary Fig. 13a–c). Similar effects were observed on actin filaments in septin 9_del1 expressing Huh7 cells (Supplementary Fig. 13d). Taken together, these results indicate the fundamental role of septin 9 and PtdIns5P in LD accumulation and cytoskeleton dynamic independently of HCV infection.

### Septin 9/PtdIns5P regulates sodium oleate-induced LD growth

**Figure 9:**
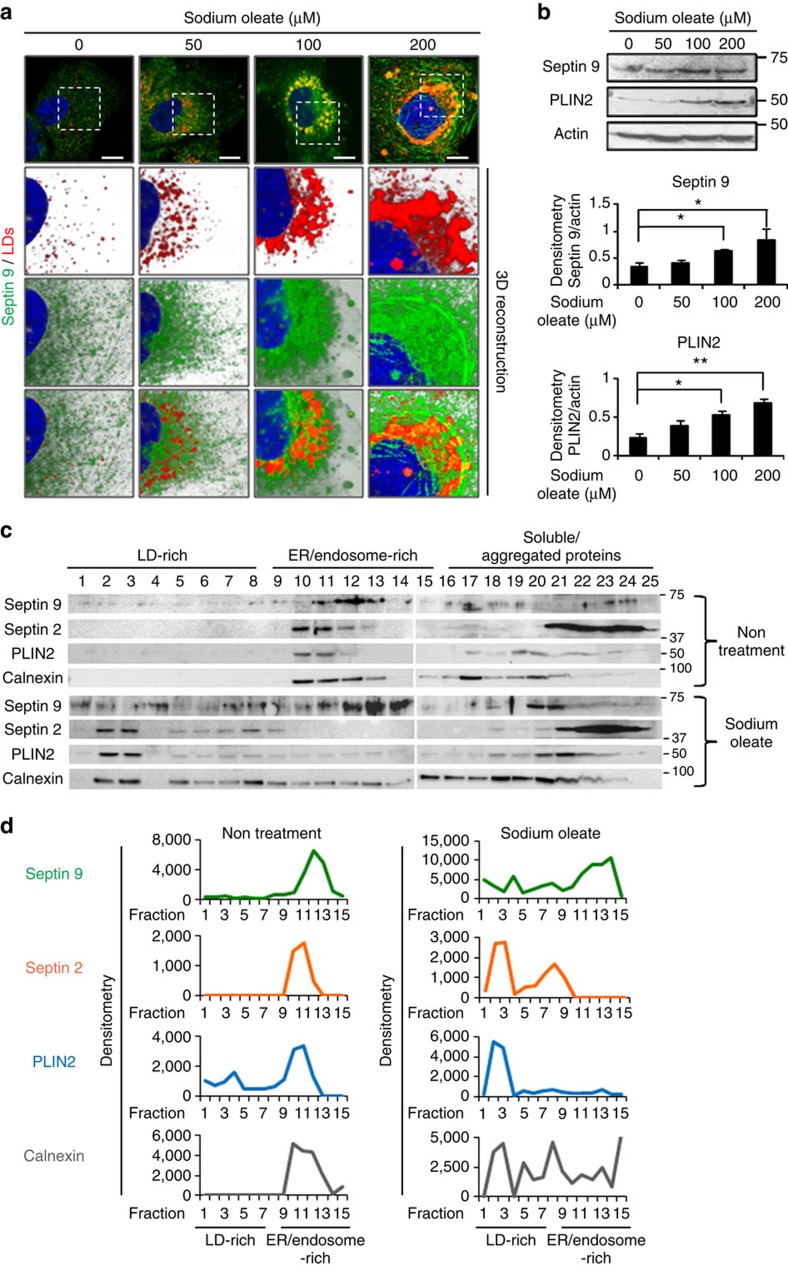
Sodium oleate treatment increases septin 9 and LDs in the perinuclear region and in LD-rich fractions. (**a**) Huh7 cells were grown for 24 h. The culture medium was supplemented with 0, 50, 100 or 200 μM sodium oleate complex for 24 h then stained for septin 9 (green) and LDs (red). The white squares indicate the zoomed area shown as a 3D reconstruction image with a white background. (**b**) Immunoblot analysis of septin 9 and PLIN2 in cells treated as in **a**. Bar graphs below show the analysis from three independent experiments. (**c**) Huh7 cells were treated or not with sodium oleate at 100 μM were submitted to membrane flotation assay and analysed by western blot for septin 9, septin 2, PLIN2 and Calnexin. (**d**) Densitometry analysis of protein expression profile from western blot in **c** is shown. Values are means±s.e.m. Student's *t*-test was used. *P<0.05, **P<0.001. Scale bar, 10 μm.

To emphasize the function of septin 9 and PtdIns5P in lipid homoeostasis, Huh7 cells were treated with sodium oleate (0–200 μM), commonly used to induce LD formation. Neutral lipid analysis revealed a concentration-dependent increase of TAG and a decrease of DAG due to the addition of sodium oleate, validating the effect of sodium oleate (Supplementary Fig. 14). The immunofluorescence data indicated a dose-dependent enlargement of the LDs and an increase of endogenous septin 9. Remarkably, in cells supplemented with 200 μM sodium oleate, septin 9 filaments were visible around the very large clusters of LDs (Fig. 9a). Immunoblot data confirmed the gradual increase of septin 9 expression with the sodium oleate concentration and a concomitant increase of PLIN2 expression (Fig. 9b).

**Figure 10:**
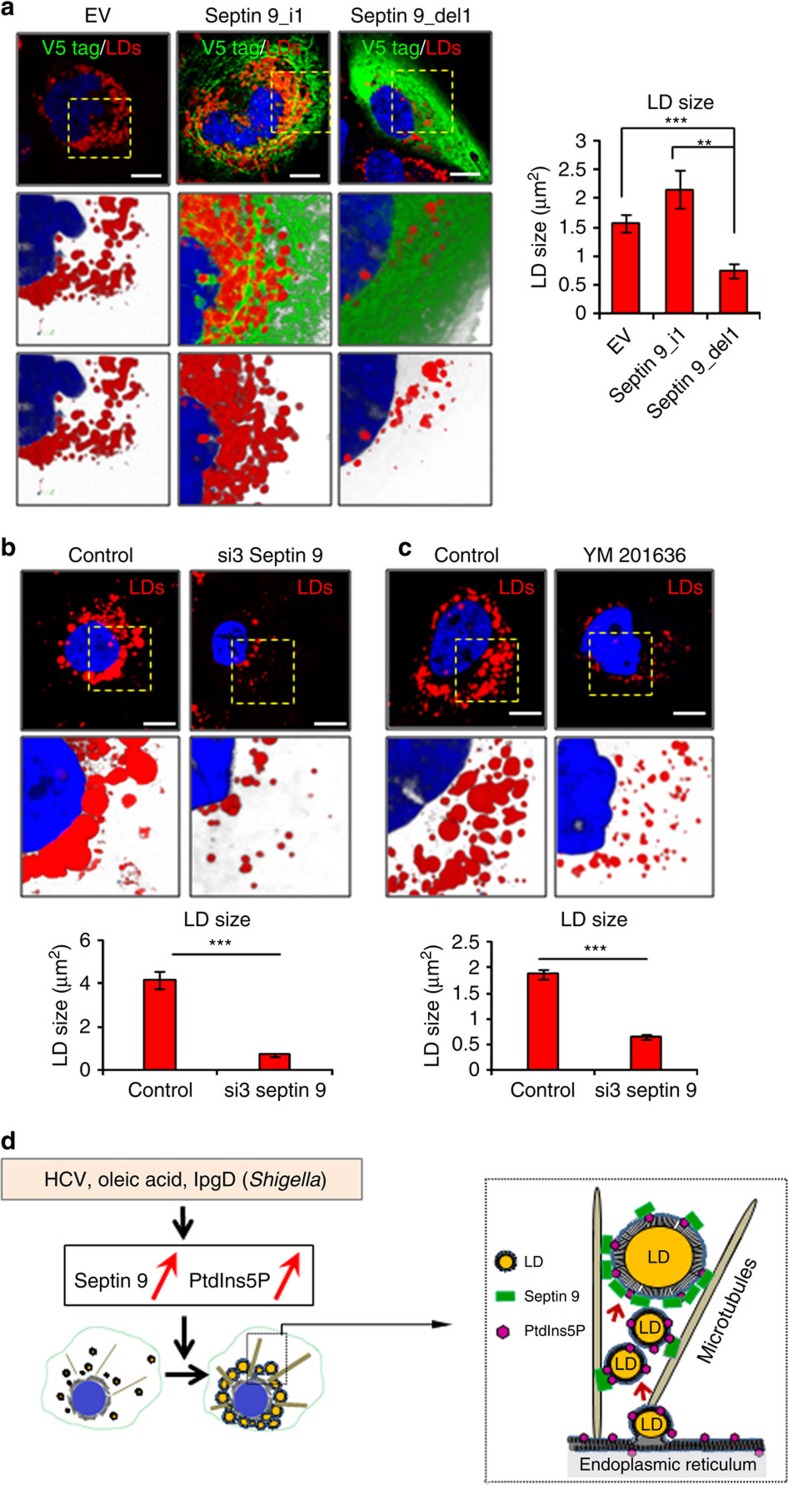
Septin 9 and PtdIns(5)P are required for LDs accumulation in Huh7 sodium oleate-treated cells. (**a**) Huh7 cells transfected with EV, septin 9_i1, or septin 9_del1 and treated with 100 μM sodium oleate complex were strained for LDs and V5 tag. Dot squares indicates the zoom area shown below in 3D reconstruction images. Bar graph shows LD size analysis of 33 cells from two independent experiments. (**b**) Huh7 cells transfected with non-targeting (control) or septin 9 siRNA (si3) were treated with 100 μM sodium oleate complex then strained for LDs and V5 tag. Bar graph shows LD size analysis of 30 cells from two independent experiments. (**c**) Huh7 cells treated with 100 μM sodium oleate then with 160 μm YM201636 for 1 h before staining for LDs (red). Bar graphs present mean LD size of 60 cells from two independent experiments. Values are means±s.e.m. Student's *t*-test was used. **P<0.001, ***P<0.0001. Scale bar, 10 μm. (**d**) Proposed model: HCV infection, oleic acid treatment and IpgD, the virulence factor of *Shigella flexneri* may increase the cellular level of both septin 9 and PtdIns5P promoting their interaction and the subsequent organization of MTs to control LD growth and accumulation.

To further investigate the association of septin 9 with LD formation, we performed membrane flotation assays^37^ (Fig. 9c,d) using Huh7 cells and the same cells treated with 100 μM sodium oleate as described in methods. Twenty-five fractions were collected and assessed by western blotting for septin 9, septin 2. PLIN2 and calnexin were analysed as markers of LDs and ER, respectively. The lowest-density membrane fractions (fractions 1–8) are called ‘LD-rich' and the fractions (9–15) represented the ‘ER/endosome-rich'fractions, while the fractions (16–25) contained soluble and aggregated proteins. In non-treated Huh7 cells, septin 9 was mainly found in the ER/endosome-rich and the soluble/aggregated protein fractions, but not within low-density LD-rich fractions (Fig. 9c, upper panels). Similar distribution profiles were observed for septin 2 and calnexin, while PLIN2 was detectable in ‘LD-rich' fractions. These data suggested that, in non-treated cells, septins are mainly associated with LDs in the ER. This distribution changed in the presence of sodium oleate, septin 9 and septin 2 increased in low-density LD-rich fractions with high expression of PLIN2 and decreased from the ER/endosome-rich fractions totally depleted of PLIN2. There was no visible change in the distribution profile of the different proteins among the soluble/aggregated protein fractions. Overall, these results suggested that septin 9 and septin 2 are associated with the ER site of LD biogenesis and sodium oleate promoted LD biogenesis and enhanced septins association with LDs. Subsequently, we explored the role of septin 9 and PtdIns5P on oleate-induced LD accumulation. Transfection with septin 9_i1 cDNA of Huh7 cells treated with 100 μM sodium oleate increased the LD size, while transfection with the septin 9_del1 construct or with si3 septin 9 (Fig. 10a,b) and YM201636 treatment (Fig. 10c), produced the opposite effect, indicating that septin 9 and PtdIns5P regulated oleate-induced LD biogenesis.

## Discussion

In this study, we showed a deregulation of septin expression in HCV-induced cirrhosis compared with that in the normal liver. Subsequent studies using HCV-infected cells revealed an unexpected role for septin 9 in the control of cellular lipid homoeostasis through its binding to PtdIns5P required for microtubules stabilization and LD perinuclear accumulation. As depicted in our model in Fig. 10d this new role of septin 9 was also observed independently of the presence of HCV in naïve cells and in sodium oleate-induced LD accumulation. Additionally, septin 9 regulates HCV replication and assembles around the perinuclear clusters of the core in HCV-infected cells. Septin 9 promotes the perinuclear recruitment of proteins coating the LD surface such as PLIN2 and PLIN3 as well as the HCV core and the non-structural NS5A. Furthermore, PLIN3 playing an essential role in the HCV replication process^37^.Thus, one could speculate that HCV recruits septin 9 to control LD growth and creates a lipid-enriched environment, facilitating HCV replication. Septins are crucial cytoskeletal components that are exploited by many pathogens such as *S. flexneri*, *Listeria monocytogenes*^23^, *Candida albicans*^50^ and *Chlamydia trachomatis*^27^. Moreover, septin 6 controls HCV replication via a direct interaction with a RNA binding protein, acting as a scaffold protein to facilitate the positioning of the HCV replication complex^29^. However, the roles of the LDs were not investigated in these studies related to septin/pathogen interactions. Here we demonstrated that IpgD, the virulence effector of *S. flexneri* increased the cellular content of PtdIns5P and induced LD growth. Even though this result confirmed the role of PtdIns5P in LD growth and accumulation, its enabled speculation on the potential role of LDs in infection by *S. flexneri*. Therefore, the findings of this study can be extended to other types of pathogens beyond HCV.

Despite the numerous publications about LD biology, several questions related to their biogenesis, growth and dynamics remain unanswered^1^,^4^,^44^. LD formation likely occurs at the ER, and different mechanisms are involved in LD growth, including growth from nascent LDs in the ER or in the vicinity of the ER via the incorporation of TAGs and phospholipids locally synthesized into LDs. These proposed mechanisms arose from the observations that key enzymes of phospholipid and neutral lipid synthesis are present on the LD surface^44^. LDs might also expand via fusion of existing LDs. These fusion processes include the absorption of small LDs by large LDs mediated by Fsp27 (a 27 kDa fat-specific protein) enriched at the contact site between LDs^51^,^52^. Fusion mechanisms can also be mediated via phospholipid metabolizing enzymes that produce fusogenic compounds at the LD surface^53^,^54^.This fusion between LDs involves the SNARE proteins^55^. In this study, we demonstrated that septin 9 significantly regulates the cellular content of TAG, a neutral lipid component essential for LD biogenesis. Furthermore, septin 9 promotes perinuclear accumulation of neutral lipid synthesis enzymes such as DGAT1 and regulates the expression of PLIN2 and PLIN3, two PLINfamily proteins crucial for LD formation. These data support a role of septin 9 in LD growth and biogenesis.

Nonetheless, we cannot rule out a role for septin 9 in LD fusion mechanisms. LD fusions are MT-dependent processes^42^. In this study, we demonstrated the requirement for dynamic MTs for septin 9-induced LD growth. Furthermore, LD fusion and size is related to the composition of the surrounding monolayer of phospholipids, including PtdIns. Interestingly, we demonstrated that septin 9 binds to mono-phosphorylated PtdIns, which is consistent with previously published data for *Saccharomyces cerevisiae*^49^. These PIs promote LD growth and are reminiscent of the role of PtdIns4P in the homoeostasis of LDs in yeasts^56^,^57^.

PtdIns5P is mainly present in the plasma and intracellular membranes, including the smooth ER and Golgi^58^, and remains the least characterized among the eight different PIs^59^. Notably, PtdIns5P regulates septin 9 assemblies in high-order structures. PtdIns5P partly rescued the loss of microtubule filaments induced by septin 9 knockdown. These results are consistent with the reported role for PIKfyve in microtubule-dependent vesicular trafficking^60^ and strongly indicate a new role for PtdIns5P as a partner of septin 9 in the regulation of MT and LD dynamics, providing new insights on PtdIns5P biological role. Consequently, we proposed that septin 9 could act as a scaffold that establishes contacts between MTs and LDs via binding to PtdIns5P that coats the surface of LDs and promotes LD growth and intracellular movement.

These results revealed an unprecedented role for PIs in LD biology in mammals, although a detailed mechanism awaits further investigations. PIs are important regulators of membrane trafficking in coordination with small GTPase proteins such as ARF GTPase, Rab GTPases, and the subunits of the COPI complex. These different components are present on the LD surface^61^,^62^. In addition, LDs interact with different cellular organelles, including the ER, Golgi and mitochondria^63^. Therefore, the existence of a different pool of LDs with a specific PI and Rab GTPase composition could be postulated and might help define LD identity as for endosomal compartments^64^. Thus, a study of the contribution of PI signalling to LD biogenesis and growth is of great interest in LD biology.

In conclusion, this study provides new insights for research on the increasing pathologies associated with lipid metabolism disorders and cancer.

## Methods

### Chemicals

Phosphoinositides (PtdIns4P diC8 Cat#P4008, PtdIns5P diC4 Cat#P5004, PtdIns3P diC8 Cat#P3008, PtdIns(4,5)P2 diC8 Cat#P4508) and PIP strip Cat#P-6001 were purchased from Echelon. Nocodazole Cat#487928 from Calbiochem, YM201636 Cat#sc-204193 from Santa Cruz. Sodium oleate Cat#o-7501 and Isopropyl β-D-1-thiogalactopyranoside (Cat# I6758) were purchased from Sigma-Aldrich. Albumin BSA FFA Cat#126575 was from Calbiochem. Purified septin 9-GST isoform 3 Cat# H00010801-P01 was purchased from Clinisciences. Purified GST was from GenScript and V5 peptide control for V5 antibody was from Novus Biologicals.

### Cell lines and culture conditions

Human hepatocarcinoma cells Huh7.5, Huh7 and stable Huh7 cells expressing HCV genomic replicon (Huh7R) were used^35^. Cells were maintained in Dulbecco's modified Eagle's medium (DMEM; Invitrogen) containing 4.5 g l^−1^ glucose and supplemented with 10% heat-inactivated fetal bovine serum, 1% nonessential amino acids (GibcoBRL) and 1% penicillin/streptomycin (GibcoBRL). For Huh7R the described medium was supplemented with G418 at 400 μg ml^−1^. Cells were tested for mycoplasma contamination weekly.

### Cell treatments

Cells treatment with sodium oleate: complexes of sodium oleate with BSA were prepared as previously reported^65^. Briefly, the solution containing 20 mM sodium oleate and 2.4 mM bovine serum albumin-fatty acid free (BSA FFA) was heated to 55 °C. The obtained complex was diluted in a pre-warmed culture medium at indicated concentration before addition to the cells. The treated cells were maintained in culture for 24 h and collected either for immunoblot or immunofluorescence experiments.

Cells treatment with YM201636: cells were incubated for 1 h at 37 °C in the pre-warmed culture medium containing 160 μM of YM201636 and collected for analyses.

Cells treatment with PIs: PIs as a lyophilized powder were solubilized at 500 μM in water by vortex. The solution was then diluted in Dulbecco's phosphate-buffered saline (DPBS) to the concentration indicated in the legends of the experiment. Cells were washed with DPBS and the PIs solution was added to the cell for indicated time.

### JFH-1/HCVcc production and inoculation

Huh7.5 were infected with cell culture-grown HCV JFH-1 at a multiplicity of infection (MOI) of 0.5 TCID_50_ cell^−1^ (50% tissue culture infective doses). After incubation at 37 °C for 16 h, the inoculum was removed then cells were washed three times with DMEM and were further maintained in culture for the indicated time in the legend and text.

### Reverse transcription and real-time PCR analysis

Total RNA was isolated using RNAble, solution (Eurobio) and HCV RNA copy number was quantified using the SuperScript III Platinium One-Step quantitative RT–PCR system (Invitrogen) and a Ligth Cycler Fast Start DNA MasterPlus SYBR Green I mix (Roche). PCR was carried on a Light Cycler 480 Real-Time PCR System (Roche). The sequence of the Primer used in real-time RT–PCR analysis was presented in Supplementary Tables 1 and 6-FAM-CCTTGTGGTACTGCCTGA-MGB (Applied Biosystems, Foster City, CA, USA) was used as internal probe. To analyse the septin 9 transcript variants, total RNA was isolated using RNeasy Mini Kit 50 (Cat# 74104 QIAGEN) and applied to reverse transcription using RevertAid First Strand cDNA Synthesis Kit (Cat#K1622 Fermentas). The cDNA was analysed by qPCR using QuantiTect SYBR Green PCR Kit (Cat#204143 QIAGEN) and a 7500 Fast Real-Time PCR System (Applied Biosystems). Reaction parameters were 30 min at 50 °C, 15 min at 95 °C, followed by 45 cycles of 15 s at 94 °C, 30 s at 55 °C and 30 s at 72 °C. The triplicate mean values were calculated using GAPDH gene transcription as reference for normalization. Used qRT–PCR primers sequences are presented in the Supplementary Table 1.

### siRNA, plasmids and transfection reagents

siRNAs from septin 9 were a Stealth RNAi siRNA (si1: Cat#SEPT9HSS173895, si3: Cat# SEPT9HSS173897) from Invitrogen and non-targeting siRNA (Cat#sc-37007) was from Santa Cruz. The sequences targeting septin 9 (si1 and si3) are presented in the Supplementary Table 2.

The cDNA of septin 9 isoform 1 transcript within pcDNA3.1/V5-His-TOPO vector (cDNA septin 9_i1) was a gift from Dr Russell (CCRCB, Queens University Belfast). The pET21d vector was a gift from Dr Pierre Nioche (INSERM UMR-S 1124, University of Paris Descartes).

cDNA septin 9_i1 vector was used as a template to generate pcDNA V5 septin 9_del1 using the QuikChange II XL Site-Direced Mutagenesis Kit (Cat#200521) from agilent following the manufacturer's recommendations. Therefore the septin 9_i1 V5/His tag and septin 9_del1 V5/His sequences have been amplified by PCR from their pcDNA3.1/V5-His-TOPO vector. The resulting DNA fragments have been inserted in a pET21d vector using In-Fusion HD Cloning System Cat#639648 (Clontech) pET21d in-frame with the C-terminal 6-histidine coding sequence of the vector and according to manufacturer recommendations to obtain pET21d septin 9_i1 V5/His and PET21d septin 9_del1 V5/His. All the primers sequences were presented in the Supplementary Table 1.

The cDNA of GFP-IpgD was a gift from Hélène Tronchère (INSERM UMR-S 1048, I2MC and Université Paul Sabatier).

The transfection of cDNA and siRNA were performed using X-tremeGENE 9 DNA Transfection Reagent (Roche Diagnostics) and Lipofectamine RNAiMAX reagent (Invitrogen) respectively following the manufacturer's protocol. The transfection was performed for 48 h unless indicated otherwise in the text.

### Protein production and purification

*E. coli* BL21(DE3) Rosetta cells (#70953-3 Novagen) were transformed with pET21d septin 9_i1 V5/His and PET21d septin 9_del1 V5/His plasmids and all culture media contained 100 μg ml^−1^ ampicillin, 34 μg ml^−1^ chloramphenicol. Cells from a single colony were used to seed an overnight 20 ml preculture of LB medium. One litre LB medium cultures from these precultures were grown at 37 °C with shaking to an optical density (OD_600 nm_) of 0.9. The temperature was brought down to 28 °C and protein expression was then induced with 1 mM Isopropyl β-D-1-thiogalactopyranoside (IPTG) from Sigma (Cat# I6758) for 4 h. The cells were harvested by centrifugation and the pellet was stored at −80 °C until used. The cell pellet was suspended in 15 ml of 50 mM sodium phosphate pH 7.4, 300 mM sodium chloride, 10% glycerol, 20 mM imidazole, 0.1% Triton X-100, and one protease inhibitor cocktail EDTA free tablet (Roche). Cell lysis was performed by sonication on ice and the cell lysate was clarified by centrifugation for 30 min at 4 °C to 40,000*g*. After filtering the supernatant the protein was isolated on a 1 ml metal affinity column (HisTrap HP, GE Healthcare) pre equilibrated in 50 mM sodium phosphate pH 7.4, 300 mM sodium chloride, 10% glycerol, 20 mM imidazole and eluted with 50 mM sodium phosphate pH 7.4, 300 mM sodium chloride, 300 mM imidazole. The fractions containing the protein of interest were further purified by cation exchange chromatography. The pooled fractions were diluted with a 1:5 ratio to a final solution of 30 mM tris pH8, 100 mM NaCl, 1 mM EDTA and then incubated with 1 ml of a strong cation exchange resin (Macro-Prep 25S, BioRad). The resin was packed in a column and eluted with a 100–600 mM NaCl linear gradient. Fractions containing the majority of protein were obtained ∼350 mM NaCl. Septins were then flash-frozen and stored at −80 °C.

### Primary antibodies

Anti septin 9 Cat#ab38314 (WB:1/500, IF:1/25), anti HCV core Cat#ab2740 (WB:1/1,000, IF:1/100), anti αtubulin Cat#ab15246 (WB:1/1,000, IF:1/100), anti septin 2 Cat#ab88657(WB:1/500, IF:1/50), mouse and rabbit anti-V5 tag Cat#ab27671 (WB:1/1,000, IF:1/400), Cat#ab9116 (WB:1/1,000, IF:1/400) respectively and anti ADFP/PLIN2 Cat#ab52355 (WB:1/2,000, IF:1/100) were from abcam; anti βtubulin Cat# T4026 (WB:1/1,000, IF:1/100) and anti TIP47/PLIN3 Cat#HPA006427 (WB:1/1,000, IF:1/100) from Sigma-Aldrich; anti-Actin Cat#sc-1616 (WB:1/1,000) and anti-PLIN2 Cat#sc-32450 (WB:1/250, IF:1/25), anti DGAT1 Cat#sc-32861 (IF:1/100) from Santa Cruz Biotechnology, anti HCV NSA Cat#HCM-131-5 (WB:1/1,000, IF:1/100) from amsbio. The mouse antibody to HCV envelope protein E2 (WB:1/500) was a gift from Dr Jean Dubuisson (CIIL, Lille, France)^66^.

### Secondary antibodies and dyes

Anti mouse IgG-HRP and anti rabbit IgG-HRP form GE healthcare (WB:1/1,000). Anti-goat IgG-HRP Cat#sc-2020 (WB:1/1,000) from Santa Cruz. Alexa Fluor 633 Cat#A21136, A21070 and A21082 (IF:1/100), Alexa Fluor 568 Cat#A11004, A11011 and A11057 (IF:1/100), Alexa Fluor 488 Cat#A11001, A21206 and A21202 (IF:1/100), streptavidin Alexa-488 Cat#S32354 (IF:1/100), streptavidin Alexa-568 Cat#S11226 (IF:1/100). Actin was stained with Alexa Fluor 594 phalloidin Cat#a12381 (IF:1/100) and nuclei with Hoechst Cat#H21486 (IF:1/5,000), both are from Invitrogen. Lipophilic fluorescence dye LD 540 (IF:1/100) from Christoph Thiele (Bonn University, Germany), was used to stain lipid droplets^67^.

### GST-2XPHD preparation

The GST-2XPHD probe was produced in BL21 RIL+ bacteria strain (Stratagene) and purified with glutathione Sepharose 4B (Amersham). After dialysis in PBS overnight and concentration with Vivaspin 500, MCO 10000, the recombinant protein was biotinylated using the BiotinTag Micro biotinylation kit (Sigma-aldrich) according to the manufacturer.

### Immunofluorescence

Cells were grown on coverslips, fixed with paraformaldehyde 4% for 20 min and permeabilized for 20 min at 37 °C using the permeabilizing buffer (PFS): DPBS containing saponin (Cat#10294440 Fisher scientific) 0.025% m.v^−1^, gelatin from cold water fish skin (Cat#G7041 Sigma 0.7% m.v^−1^). Then cells were incubated with primary antibody for 2 h washed three times for 5 min with PFS and incubated with the appropriate secondary antibodies or with the dye for 90 min. The coverslips were mounted using Prolong Gold (Cat#P36934 Invitrogen). For PtdIns5P staining the cells on coverslips were fixed and permeabilized as described above and then incubated with recombinant Bio-GST-2XPHDING2 (prepared as describe above) in 1% BSA-PBS for 1 h. Following incubation, cells were washed and incubated for another hour with streptavidin Alexa-488 (1/300) (Invitrogen) and Hoechst for nuclei staining, mounted and analysed for immunofluorescence.

### Images acquisition and analysis

Images acquired with a Leica TCS SP5 AOBS tandem confocal microscope were analysed by Icy bioimage analysis software for 3D reconstruction. For colocalization analysis, images were treated by ImageJ software, the plugin ‘Intensity Correlation Analysis' was used to generate the Pearson's correlation coefficient (Rr) which range from −1 (perfect exclusion) to +1 (perfect correlation).

To determine LD distribution in cells, the plugin ‘Radial profil' was used. For analysis, a circle was defined at the periphery of each cell and the plugin produces a profile plot of normalized integrated intensities around concentric circles as a function of distance from a point in the image, considered here as the centre of the cell. The concentric circles were assembled in three circle bands, the first corresponding to the area of the nuclei and the rest corresponding to the cytoplasm was divided in two equal bands (the band near the nuclei is considered as the ‘perinuclear' and the other the ‘periphery'). The intensity in each band was calculated from the total integrated intensities around concentric circles present in the band.

To calculate the size and number of LDs, images obtained by confocal microscopy were processed by background subtraction and standardized thresholding (default), cell ROI was made by free hand selection tool, then LDs size and number was obtained by ImageJ analyse particles function (particle area less than 0.01 μm^2^ were excluded).

### Immunoblot

Cells were washed with ice-cold DPBS and lysed on ice using the following buffer: 20 mM Tris, HCl, 100 mM NaCl, 1% Triton X-100 at PH 7.4 containing protease inhibitors (cOmplete ULTRA Cat#05892970001 Roche). The proteins were separated on SDS–PAGE and electrotransferred onto nitrocellulose membrane. After transfer, the membrane was saturated in DPBS containing 0.1% Tween 20 and 5% milk. Primary antibodies were added overnight at 4 °C or for 2 h at room temperature depending on the antibody. The membranes were washed with DPBS and incubated for 1 h at room temperature with appropriate secondary antibody coupled with peroxidase. ECL plus kit (Cat#32132 Thermo Scientific) was used for protein detection. Chemiluminescent signal was detected by G:BOX Chemi Fluorescent & Chemiluminescent Imaging System from SYNGENE. Blot quantification was done using ImageJ software.

Blot Scans are presented in the figures and the uncropped scan are supplied in Supplementary Fig. 15.

### PIP strip overlay assay

Membranes PIP Strips (Echelon Biosciences) were blocked with 3% BSA FFA dissolved in phosphate-buffered saline (PBS) containing 0.1% Tween 20 (3% BSA FFA PBS-T) at room temperature for 60 min, then incubated overnight at 4 °C with the same buffer containing the purified protein of interest at a concentration of 0.5 μg ml^−1^ or the purified corresponding tag (V5 or GST) at equivalent molar concentration used as controls. The membranes were washed and bounded proteins were detected with suitable antibody.

### Neutral lipids analysis

This analysis was performed using the facility service of Metatoul in Toulouse (http://www.metatoul.fr). One million of cells were used for lipid extraction according to Bligh and Dyer^68^ using dichloromethane/methanol/water (2.5:2.5:2.1, v/v/v) mix, in the presence of the internal standards: 4 μg of stigmasterol, 4 μg of glycerid dimyristoleate, 4 μg of cholesteryl heptadecanoate, 8 μg of glyceryl trinonadecanoate. Organic phases were collected and evaporated to dryness and neutral lipids were separated through a SPE cartridge. Briefly SiOH cartridge (200 mg, Macherey Nagel) was equilibrated with 2 ml of dichloromethane and the lipid extract was put down in 20 μl of a solution of dichloromethane containing 10% omethanol and loaded. Neutral lipids were eluted with 2 ml of the same mixture. The final extract were concentrated and dissolved in 20 μl of ethyl acetate. One microlitre of the lipid extract was analysed by gas-liquid chromatography on a FOCUS Thermo Electron system using an Zebron-1 Phenomenex fused silica capillary columns (5 m × 0,32 mm i.d., 0.50 μm film thickness)^69^. The temperature of the Oven was programmed from 200 to 350 °C at a rate of 5 °C per min and the carrier gas was hydrogen (0.5 bar). The injector and the detector were at 315 and 345 °C, respectively.

### Membrane flotation assay

The assay was performed as recently reported^37^. Huh7 cells treated with 100 μM sodium oleate for 24 h were trypsinized, washed and counted. Cells (15 × 10^6^) total for each sample were resuspended in 1.75 ml PBS-containing 0.25 M sucrose (PBS/sucrose) supplemented with protease inhibitor cocktail (Sigma). The cells were then lysed with 200 passages in a tight fitting dounce homogenizer to ensure ∼90% lysis. The cell lysate was then spun at 2,500*g* for 10 min at 4 °C to pellet cellular debris and nuclei. Equal amounts of protein (2.5 mg) for each cell type was adjusted in volume to 1 ml with PBS/sucrose and mixed with 1 ml of 60% iodixanol (Sigma) resulting in a 30% iodixanol concentration. A discontinuous iodixanol gradient (10%, 20%) was layered on top of the lysate/iodixanol mixture, and the gradient was spun at 200,000*g* for 16 h at 4 °C in a MLS 50 Rotor. A total of 25 fractions were collected from top to bottom. The fractions were mixed 1:1 with Laemmli buffer for immunoblot.

### Transcriptome analysis

Transcriptome data set GSE14323 (ref. 31) was downloaded from NCBI website (http://www.ncbi.nlm.nih.gov/geo/query/acc.cgi?acc=GSE14323): normalized matrix by users (RMA method in the R affy package) was employed to realize analysis. This data set is composed of 19 control samples of normal liver and 49 samples of cirrhosis. R software version 3.2.2 was employed to perform, principal component analysis, boxplots, heatmap, two-sided Student *t*-test, also algorithm of random forest (RF). The learning machine application RF was performed by initializing the function tuneRF to determine the mtry parameter with the optimal error of bag. The algorithm was implemented with a learning based on 500 classification trees with the optimal mtry parameter previously determined^70^.

### Statistical analyses

Unpaired Student's *t*-tests was used and statistical significance was determined at *P<0.05; **P<0.001, ***P<0.0001.

### Data availability

The data that support the findings of this study are available from the corresponding author upon request. Transcriptome data set GSE14323 (ref. 31) was downloaded from NCBI website (http://www.ncbi.nlm.nih.gov/geo/query/acc.cgi?acc=GSE14323).

## Additional information

**How to cite this article:** Akil, A. *et al*. Septin 9 induces lipid droplets growth by a phosphatidylinositol-5-phosphate and microtubule-dependent mechanism hijacked by HCV. *Nat. Commun.* 7:12203 doi: 10.1038/ncomms12203 (2016).

## Supplementary Material

Supplementary Information Supplementary Figures 1-15 and Supplementary Tables 1-2

## References

[b1] Pol A., Gross S. P. & Parton R. G. Review: biogenesis of the multifunctional lipid droplet: lipids, proteins, and sites. J. Cell Biol. 204, 635–646 (2014). 24590170 10.1083/jcb.201311051 PMC3941045

[b2] Welte M. A. Expanding roles for lipid droplets. Curr. Biol. 25, R470–R481 (2015). 26035793 10.1016/j.cub.2015.04.004 PMC4452895

[b3] Walther T. C. & Farese R. V. Lipid droplets and cellular lipid metabolism. Annu. Rev. Biochem. 81, 687–714 (2012). 22524315 10.1146/annurev-biochem-061009-102430 PMC3767414

[b4] Ohsaki Y., Suzuki M. & Fujimoto T. Open questions in lipid droplet biology. Chem. Biol. 21, 86–96 (2014). 24239006 10.1016/j.chembiol.2013.08.009

[b5] Bickel P. E., Tansey J. T. & Welte M. A. PAT proteins, an ancient family of lipid droplet proteins that regulate cellular lipid stores. Biochim. Biophys. Acta 1791, 419–440 (2009). 19375517 10.1016/j.bbalip.2009.04.002 PMC2782626

[b6] Yamane D., McGivern D. R., Masaki T. & Lemon S. M. Liver injury and disease pathogenesis in chronic hepatitis C. Curr. Top. Microbiol. Immunol. 369, 263–288 (2013). 23463205 10.1007/978-3-642-27340-7_11

[b7] Barba G. . Hepatitis C virus core protein shows a cytoplasmic localization and associates to cellular lipid storage droplets. Proc. Natl Acad. Sci. USA 94, 1200–1205 (1997). 9037030 10.1073/pnas.94.4.1200 PMC19768

[b8] Paul D., Madan V. & Bartenschlager R. Hepatitis C virus RNA replication and assembly: living on the fat of the land. Cell Host Microbe 16, 569–579 (2014). 25525790 10.1016/j.chom.2014.10.008 PMC7172941

[b9] Miyanari Y. . The lipid droplet is an important organelle for hepatitis C virus production. Nat. Cell Biol. 9, 1089–1097 (2007). 17721513 10.1038/ncb1631

[b10] Boulant S. . Structural determinants that target the hepatitis C virus core protein to lipid droplets. J. Biol. Chem. 281, 22236–22247 (2006). 16704979 10.1074/jbc.M601031200

[b11] Tauchi-Sato K., Ozeki S., Houjou T., Taguchi R. & Fujimoto T. The surface of lipid droplets is a phospholipid monolayer with a unique fatty acid composition. J. Biol. Chem. 277, 44507–44512 (2002). 12221100 10.1074/jbc.M207712200

[b12] Olofsson S.-O. . Lipid droplets as dynamic organelles connecting storage and efflux of lipids. Biochim. Biophys. Acta 1791, 448–458 (2009). 18775796 10.1016/j.bbalip.2008.08.001

[b13] Balla T. Phosphoinositides: tiny lipids with giant impact on cell regulation. Physiol. Rev. 93, 1019–1137 (2013). 23899561 10.1152/physrev.00028.2012 PMC3962547

[b14] Maehama T., Fukasawa M., Date T., Wakita T. & Hanada K. A class II phosphoinositide 3-kinase plays an indispensable role in hepatitis C virus replication. Biochem. Biophys. Res. Commun. 440, 150–156 (2013). 24055031 10.1016/j.bbrc.2013.09.048

[b15] Gassama-Diagne A. . Phosphatidylinositol-3,4,5-trisphosphate regulates the formation of the basolateral plasma membrane in epithelial cells. Nat. Cell Biol. 8, 963–970 (2006). 16921364 10.1038/ncb1461

[b16] Martin-Belmonte F. . PTEN-mediated apical segregation of phosphoinositides controls epithelial morphogenesis through Cdc42. Cell 128, 383–397 (2007). 17254974 10.1016/j.cell.2006.11.051 PMC1865103

[b17] Awad A. . SHIP2 regulates epithelial cell polarity through its lipid product, which binds to Dlg1, a pathway subverted by hepatitis C virus core protein. Mol. Biol. Cell 24, 2171–2185 (2013). 23699395 10.1091/mbc.E12-08-0626 PMC3708724

[b18] Nishihama R., Onishi M. & Pringle J. R. New insights into the phylogenetic distribution and evolutionary origins of the septins. Biol. Chem. 392, 681–687 (2011). 21824002 10.1515/BC.2011.086 PMC3951473

[b19] Weirich C. S., Erzberger J. P. & Barral Y. The septin family of GTPases: architecture and dynamics. Nat. Rev. Mol. Cell Biol. 9, 478–489 (2008). 18478031 10.1038/nrm2407

[b20] Barral Y. & Kinoshita M. Structural insights shed light onto septin assemblies and function. Curr. Opin. Cell Biol. 20, 12–18 (2008). 18242072 10.1016/j.ceb.2007.12.001

[b21] Dolat L., Hu Q. & Spiliotis E. T. Septin functions in organ system physiology and pathology. Biol. Chem. 395, 123–141 (2014). 24114910 10.1515/hsz-2013-0233 PMC4452026

[b22] Fung K. Y. Y., Dai L. & Trimble W. S. Cell and molecular biology of septins. Int. Rev. Cell Mol. Biol. 310, 289–339 (2014). 24725429 10.1016/B978-0-12-800180-6.00007-4

[b23] Mostowy S. & Cossart P. Septins: the fourth component of the cytoskeleton. Nat. Rev. Mol. Cell Biol. 13, 183–194 (2012). 22314400 10.1038/nrm3284

[b24] Bertin A. . Phosphatidylinositol-4,5-bisphosphate promotes budding yeast septin filament assembly and organization. J. Mol. Biol. 404, 711–731 (2010). 20951708 10.1016/j.jmb.2010.10.002 PMC3005623

[b25] Tanaka-Takiguchi Y., Kinoshita M. & Takiguchi K. Septin-mediated uniform bracing of phospholipid membranes. Curr. Biol. 19, 140–145 (2009). 19167227 10.1016/j.cub.2008.12.030

[b26] Zhang J. . Phosphatidylinositol polyphosphate binding to the mammalian septin H5 is modulated by GTP. Curr. Biol. 9, 1458–1467 (1999). 10607590 10.1016/s0960-9822(00)80115-3

[b27] Volceanov L. . Septins arrange F-actin-containing fibers on the *Chlamydia trachomatis* inclusion and are required for normal release of the inclusion by extrusion. mBio 5, e01802–e01814 (2014). 25293760 10.1128/mBio.01802-14 PMC4196233

[b28] Bridges A. A. & Gladfelter A. S. Fungal pathogens are platforms for discovering novel and conserved septin properties. Curr. Opin. Microbiol. 20, 42–48 (2014). 24879478 10.1016/j.mib.2014.04.004 PMC4266239

[b29] Kim C. S., Seol S. K., Song O.-K., Park J. H. & Jang S. K. An RNA-binding protein, hnRNP A1, and a scaffold protein, septin 6, facilitate hepatitis C virus replication. J. Virol. 81, 3852–3865 (2007). 17229681 10.1128/JVI.01311-06 PMC1866118

[b30] Dos Santos A. . Identification of cellular targets in human intrahepatic cholangiocarcinoma using laser microdissection and accurate mass and time tag proteomics. Mol. Cell. Proteomics 9, 1991–2004 (2010). 20513801 10.1074/mcp.M110.000026 PMC2938110

[b31] Mas V. R. . Genes involved in viral carcinogenesis and tumor initiation in hepatitis C virus–induced hepatocellular carcinoma. Mol. Med. 15, 85–94 (2009). 19098997 10.2119/molmed.2008.00110 PMC2605622

[b32] Kim M. S., Froese C. D., Estey M. P. & Trimble W. S. SEPT9 occupies the terminal positions in septin octamers and mediates polymerization-dependent functions in abscission. J. Cell Biol. 195, 815–826 (2011). 22123865 10.1083/jcb.201106131 PMC3257574

[b33] Wakita T. . Production of infectious hepatitis C virus in tissue culture from a cloned viral genome. Nat. Med. 11, 791–796 (2005). 15951748 10.1038/nm1268 PMC2918402

[b34] Heid H. W., Moll R., Schwetlick I., Rackwitz H. R. & Keenan T. W. Adipophilin is a specific marker of lipid accumulation in diverse cell types and diseases. Cell Tissue Res. 294, 309–321 (1998). 9799447 10.1007/s004410051181

[b35] Pietschmann T. . Persistent and transient replication of full-length hepatitis C virus genomes in cell culture. J. Virol. 76, 4008–4021 (2002). 11907240 10.1128/JVI.76.8.4008-4021.2002 PMC136109

[b36] Ploen D. . TIP47 is associated with the hepatitis C virus and its interaction with Rab9 is required for release of viral particles. Eur. J. Cell Biol. 92, 374–382 (2013). 24480419 10.1016/j.ejcb.2013.12.003

[b37] Vogt D. A. . Lipid droplet-binding protein TIP47 regulates hepatitis C Virus RNA replication through interaction with the viral NS5A protein. PLoS Pathog. 9, e1003302 (2013). 23593007 10.1371/journal.ppat.1003302 PMC3623766

[b38] Yen C.-L. E., Stone S. J., Koliwad S., Harris C. & Farese R. V. Thematic review series: glycerolipids. DGAT enzymes and triacylglycerol biosynthesis. J. Lipid Res. 49, 2283–2301 (2008). 18757836 10.1194/jlr.R800018-JLR200 PMC3837458

[b39] Herker E. . Efficient hepatitis C virus particle formation requires diacylglycerol acyltransferase-1. Nat. Med. 16, 1295–1298 (2010). 20935628 10.1038/nm.2238 PMC3431199

[b40] Camus G. . Diacylglycerol acyltransferase-1 localizes hepatitis C virus NS5A protein to lipid droplets and enhances NS5A interaction with the viral capsid core. J. Biol. Chem. 288, 9915–9923 (2013). 23420847 10.1074/jbc.M112.434910 PMC3617291

[b41] Thiam A. R., Farese R. V. & Walther T. C. The biophysics and cell biology of lipid droplets. Nat. Rev. Mol. Cell Biol. 14, 775–786 (2013). 24220094 10.1038/nrm3699 PMC4526153

[b42] Boström P. . Cytosolic lipid droplets increase in size by microtubule-dependent complex formation. Arterioscler. Thromb. Vasc. Biol. 25, 1945–1951 (2005). 16051877 10.1161/01.ATV.0000179676.41064.d4

[b43] Sellin M. E., Stenmark S. & Gullberg M. Mammalian SEPT9 isoforms direct microtubule-dependent arrangements of septin core heteromers. Mol. Biol. Cell 23, 4242–4255 (2012). 22956766 10.1091/mbc.E12-06-0486 PMC3484102

[b44] Yang H., Galea A., Sytnyk V. & Crossley M. Controlling the size of lipid droplets: lipid and protein factors. Curr. Opin. Cell Biol. 24, 509–516 (2012). 22726586 10.1016/j.ceb.2012.05.012

[b45] Gozani O. . The PHD finger of the chromatin-associated protein ING2 functions as a nuclear phosphoinositide receptor. Cell 114, 99–111 (2003). 12859901 10.1016/s0092-8674(03)00480-x

[b46] Coronas S. . Elevated levels of PtdIns5P in NPM-ALK transformed cells: implication of PIKfyve. Biochem. Biophys. Res. Commun. 372, 351–355 (2008). 18501703 10.1016/j.bbrc.2008.05.062

[b47] Niebuhr K. . Conversion of PtdIns(4,5)P(2) into PtdIns(5)P by the *S.flexneri* effector IpgD reorganizes host cell morphology. EMBO J. 21, 5069–5078 (2002). 12356723 10.1093/emboj/cdf522 PMC129044

[b48] Sbrissa D., Ikonomov O. C., Filios C., Delvecchio K. & Shisheva A. Functional dissociation between PIKfyve-synthesized PtdIns5P and PtdIns(3,5)P2 by means of the PIKfyve inhibitor YM201636. Am. J. Physiol. Cell Physiol. 303, C436–C446 (2012). 22621786 10.1152/ajpcell.00105.2012 PMC3422984

[b49] Casamayor A. & Snyder M. Molecular dissection of a yeast septin: distinct domains are required for septin interaction, localization, and function. Mol. Cell. Biol. 23, 2762–2777 (2003). 12665577 10.1128/MCB.23.8.2762-2777.2003 PMC152559

[b50] Phan Q. T. . Role of endothelial cell septin 7 in the endocytosis of Candida albicans. mBio 4, e00542–513 (2013). 24345743 10.1128/mBio.00542-13 PMC3870263

[b51] Gong J., Sun Z. & Li P. CIDE proteins and metabolic disorders. Curr. Opin. Lipidol. 20, 121–126 (2009). 19276890 10.1097/MOL.0b013e328328d0bb

[b52] Wu L. . Rab8a-AS160-MSS4 regulatory circuit controls lipid droplet fusion and growth. Dev. Cell 30, 378–393 (2014). 25158853 10.1016/j.devcel.2014.07.005

[b53] Guijas C., Rodríguez J. P., Rubio J. M., Balboa M. A. & Balsinde J. Phospholipase A2 regulation of lipid droplet formation. Biochim. Biophys. Acta 1841, 1661–1671 (2014). 25450448 10.1016/j.bbalip.2014.10.004

[b54] Andersson L. . PLD1 and ERK2 regulate cytosolic lipid droplet formation. J. Cell Sci. 119, 2246–2257 (2006). 16723731 10.1242/jcs.02941

[b55] Boström P. . SNARE proteins mediate fusion between cytosolic lipid droplets and are implicated in insulin sensitivity. Nat. Cell Biol. 9, 1286–1293 (2007). 17922004 10.1038/ncb1648

[b56] Foti M., Audhya A. & Emr S. D. Sac1 lipid phosphatase and Stt4 phosphatidylinositol 4-kinase regulate a pool of phosphatidylinositol 4-phosphate that functions in the control of the actin cytoskeleton and vacuole morphology. Mol. Biol. Cell 12, 2396–2411 (2001). 11514624 10.1091/mbc.12.8.2396 PMC58602

[b57] Ren J. . A phosphatidylinositol transfer protein integrates phosphoinositide signaling with lipid droplet metabolism to regulate a developmental program of nutrient stress-induced membrane biogenesis. Mol. Biol. Cell 25, 712–727 (2014). 24403601 10.1091/mbc.E13-11-0634 PMC3937096

[b58] Sarkes D. & Rameh L. E. A novel HPLC-based approach makes possible the spatial characterization of cellular PtdIns5P and other phosphoinositides. Biochem. J. 428, 375–384 (2010). 20370717 10.1042/BJ20100129 PMC2944655

[b59] Viaud J., Boal F., Tronchère H., Gaits-Iacovoni F. & Payrastre B. Phosphatidylinositol 5-phosphate: a nuclear stress lipid and a tuner of membranes and cytoskeleton dynamics. BioEssays News Rev. Mol. Cell. Dev. Biol. 36, 260–272 (2014). 10.1002/bies.201300132 24375703

[b60] Ikonomov O. C. . Kinesin adapter JLP links PIKfyve to microtubule-based endosome-to-trans-Golgi network traffic of furin. J. Biol. Chem. 284, 3750–3761 (2009). 19056739 10.1074/jbc.M806539200 PMC2635046

[b61] Bartz R. . Dynamic activity of lipid droplets: protein phosphorylation and GTP-mediated protein translocation. J. Proteome Res. 6, 3256–3265 (2007). 17608402 10.1021/pr070158j

[b62] Wilfling F., Haas J. T., Walther T. C. & Farese R. V. Lipid droplet biogenesis. Curr. Opin. Cell Biol. 29, 39–45 (2014). 24736091 10.1016/j.ceb.2014.03.008 PMC4526149

[b63] Barbosa A. D., Savage D. B. & Siniossoglou S. Lipid droplet-organelle interactions: emerging roles in lipid metabolism. Curr. Opin. Cell Biol. 35, 91–97 (2015). 25988547 10.1016/j.ceb.2015.04.017

[b64] Di Paolo G. & De Camilli P. Phosphoinositides in cell regulation and membrane dynamics. Nature 443, 651–657 (2006). 17035995 10.1038/nature05185

[b65] Tian Y. . Differential modulation of L-type calcium channel subunits by oleate. Am. J. Physiol. Endocrinol. Metab. 294, E1178–E1186 (2008). 18430963 10.1152/ajpendo.90237.2008 PMC2640323

[b66] Flint M. . Characterization of Hepatitis C Virus E2 Glycoprotein Interaction with a Putative Cellular Receptor, CD81. J. Virol. 73, 6235–6244 (1999). 10400713 10.1128/jvi.73.8.6235-6244.1999 PMC112700

[b67] Spandl J., White D. J., Peychl J. & Thiele C. Live cell multicolor imaging of lipid droplets with a new dye, LD540. Traffic 10, 1579–1584 (2009). 19765264 10.1111/j.1600-0854.2009.00980.x

[b68] Bligh E. G. & Dyer W. J. A rapid method of total lipid extraction and purification. Can. J. Biochem. Physiol. 37, 911–917 (1959). 13671378 10.1139/o59-099

[b69] Barrans A. . Hepatic lipase induces the formation of pre-beta 1 high density lipoprotein (HDL) from triacylglycerol-rich HDL2. A study comparing liver perfusion to in vitro incubation with lipases. J. Biol. Chem. 269, 11572–11577 (1994). 8157689

[b70] Breiman L. Random forests. Mach. Learn. 45, 5–32 (2001).

