# Supplementary material for: Septin 9 induces lipid droplets growth by a phosphatidylinositol-5-phosphate and microtubule-dependent mechanism hijacked by HCV
Source: Nat Commun. 2016 Jul 15;7:12203. doi: 10.1038/ncomms12203 (PMC4947189; doi:10.1038/ncomms12203)
Supplement: Supplementary Information — Supplementary Figures 1-15 and Supplementary Tables 1-2 [file ncomms12203-s1.pdf]

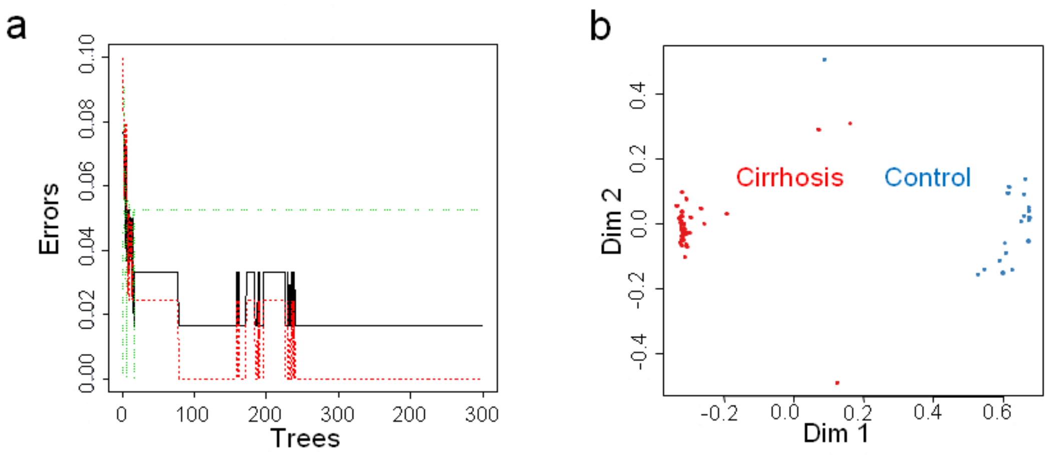

c

confusion: 1.67%	HCV cirrhosis	control	Class error
**HCV-cirrhosis**	41	0	0.00000000
**control**	1	18	0.05263158

Supplementary Figure 1: Deregulation of septins in human cirrhosis.

(a) R software with Random Forest package was used to obtain class error by “Septin” random forest learning machine on the basis of 500 trees. (b) MDSplot of individual separation by “Septin” random forest. (c) Matrix of confusion obtained on “Septin” Random Forest.

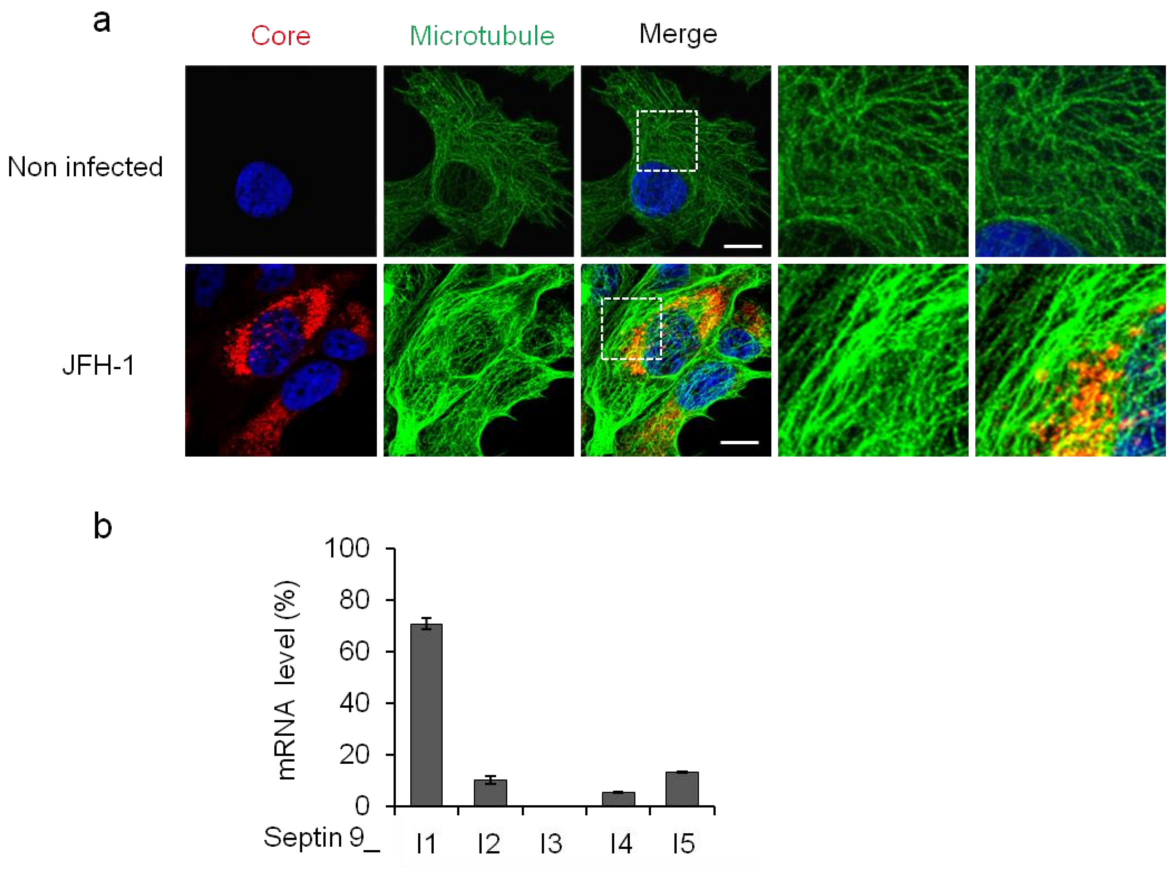

Supplementary Figure 2: Microtubules network in HCV infected cells.

(a) Huh7.5 cells were infected or not with JFH-1 for 72 h then stained for microtubules with α tubulin (green) and core (red). Scale bar, 10 µm. (b) Huh7.5 cells were infected with JFH-1 for 48 h prior to mRNA extraction and septin 9 variants (1-5) mRNA were analyzed by qRT-PCR in triplicate experiment.

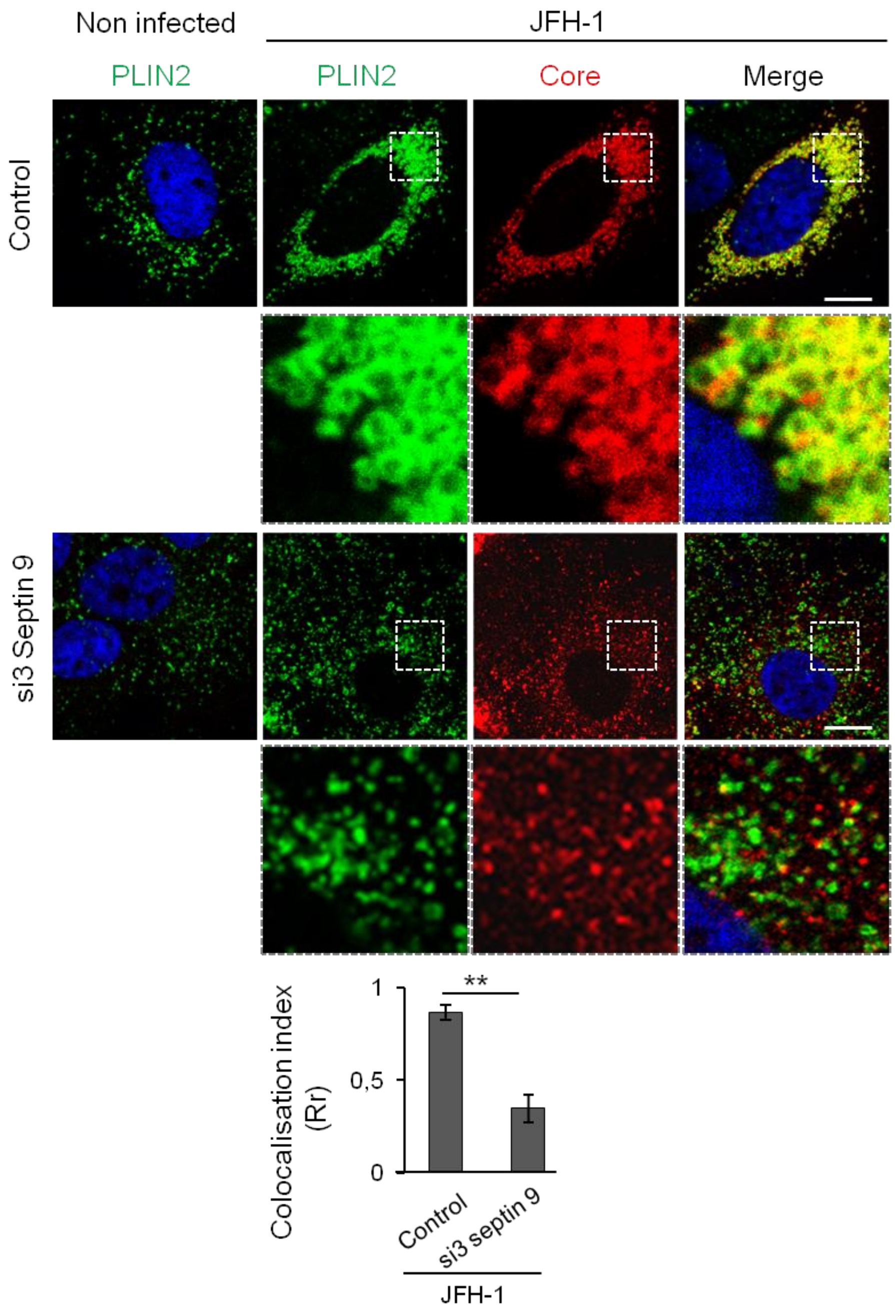

Supplementary Figure 3: Septin 9 depletion decreases PLIN2

Huh7.5 cells were transfected with non-targeting (control) or septin 9 siRNA si3 for 24 h then infected or not with JFH-1 for further 48 h prior to staining for PLIN2 (green) and core (red). Dot squares are presented in higher magnification below each panel. Scale bar, 10 µm. Bar graph represents Pearson's correlation coefficient (Rr) analysis of septin 9 and PLIN2 calculated in 20 cells from 2 independent experiments. Values are means±s.e.m. Student's *t*-test was used. $^{**}P < 0.001$.

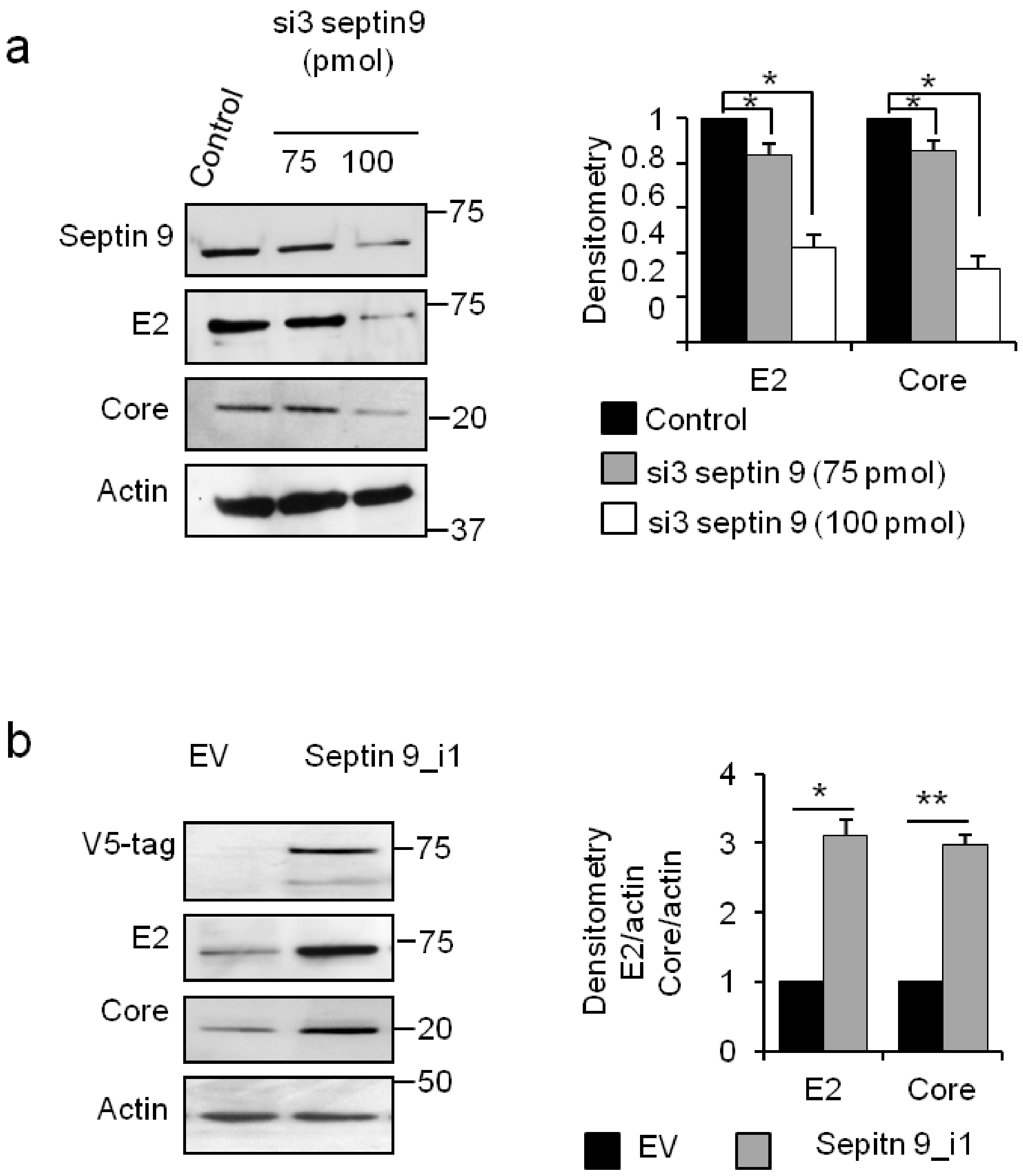

Supplementary Figure 4: Septin 9 regulates core and E2 expression in Huh7R cells

(a) Immunoblot analysis of core and E2 in Huh7R cells transfected either with 75 or 100 pmol of septin 9 siRNA si3 or with 100 pmol non-targeting (control). The bar graph presents the densitometry analysis of 3 independent experiments. (b) Immunoblot analysis of core and E2 in Huh7R cells transfected with either EV or septin 9_i1 for 48 h. The bar graph presents immunoblots analysis from 3 independent experiments. Values are means±s.e.m. Student's *t*-test was used. **P* < 0.05, ***P* < 0.001.

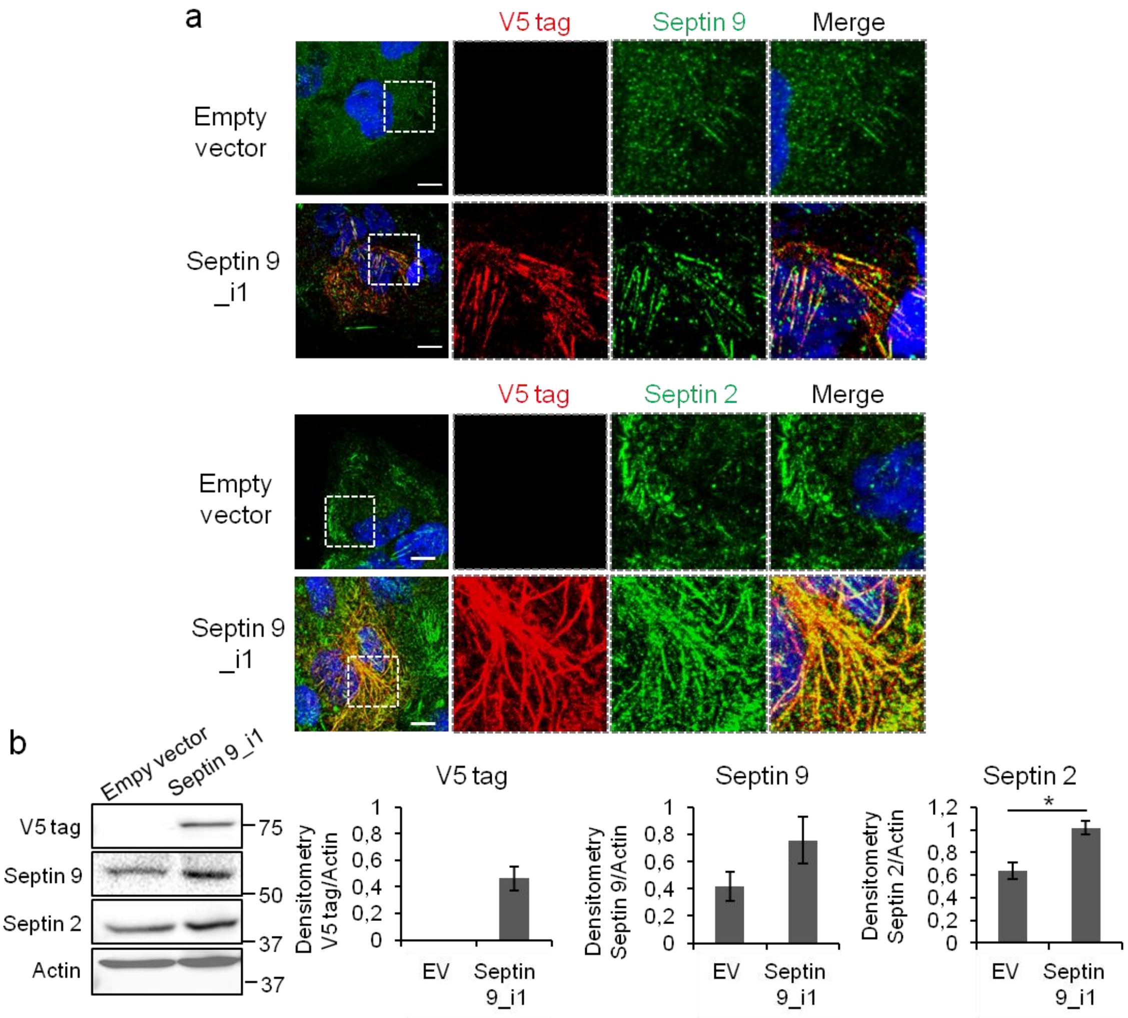

Supplementary Figure 5: Septin 9_i1 colocalizes with septin 9 and septin 2

(a) Huh7R cells were transfected with empty vector (EV) or septin 9_i1 and stained for V5 tag (red) with endogenous septin 9 (green) or with septin 2 (green). (b) Immunoblot analysis of cells in (a). Bar graphs present the densitometry analysis of detected V5 tag, septin 9 and septin 2 in 3 independent experiments. Values are means±s.e.m. Student's *t*-test was used. **P* < 0.05. Scale bar, 10 µm.

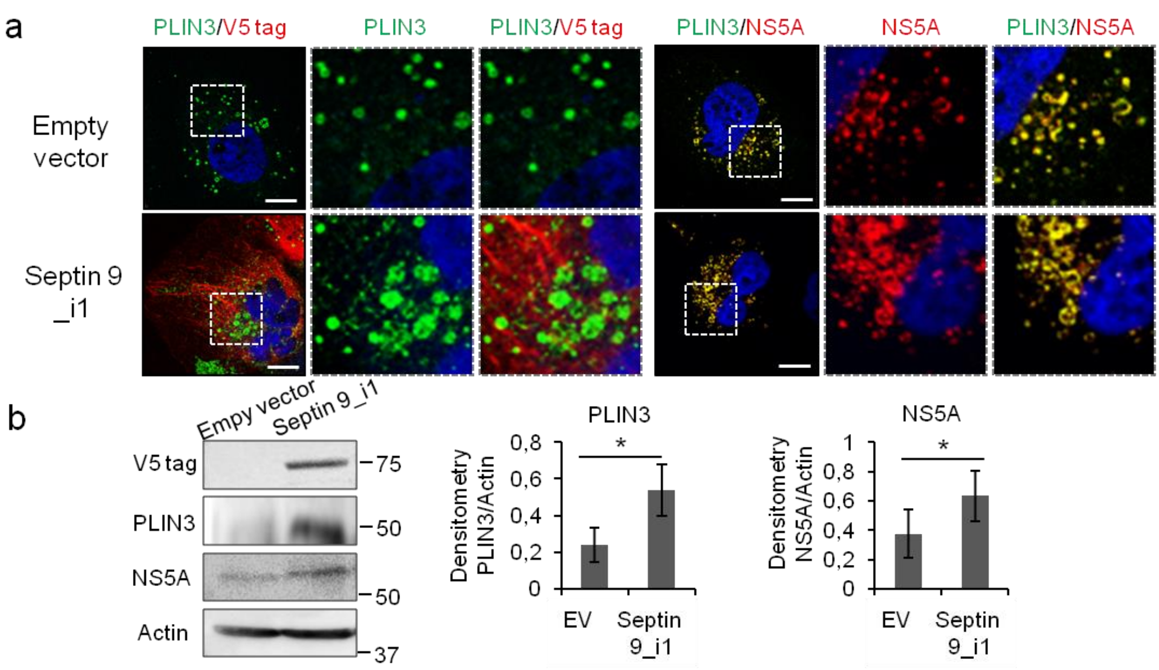

Supplementary Figure 6: Septin 9_i1 regulates PLIN3 and NS5A expression

(a) Huh7R cells transfected with either empty vector or septin 9_i1 were stained for PLIN3 (green) with V5 tag or with NS5A (red). (b) Immunoblot of NS5A and PLIN3 in Huh7R cells transfected with either empty vector or septin 9_i1. Bar graph presents the densitometry analysis of PLIN3 and NS5A in the immunoblots from 3 independent experiments. Values are means±s.e.m. Student's *t*-test was used. *$P < 0.05$. Scale bar, 10 μm.

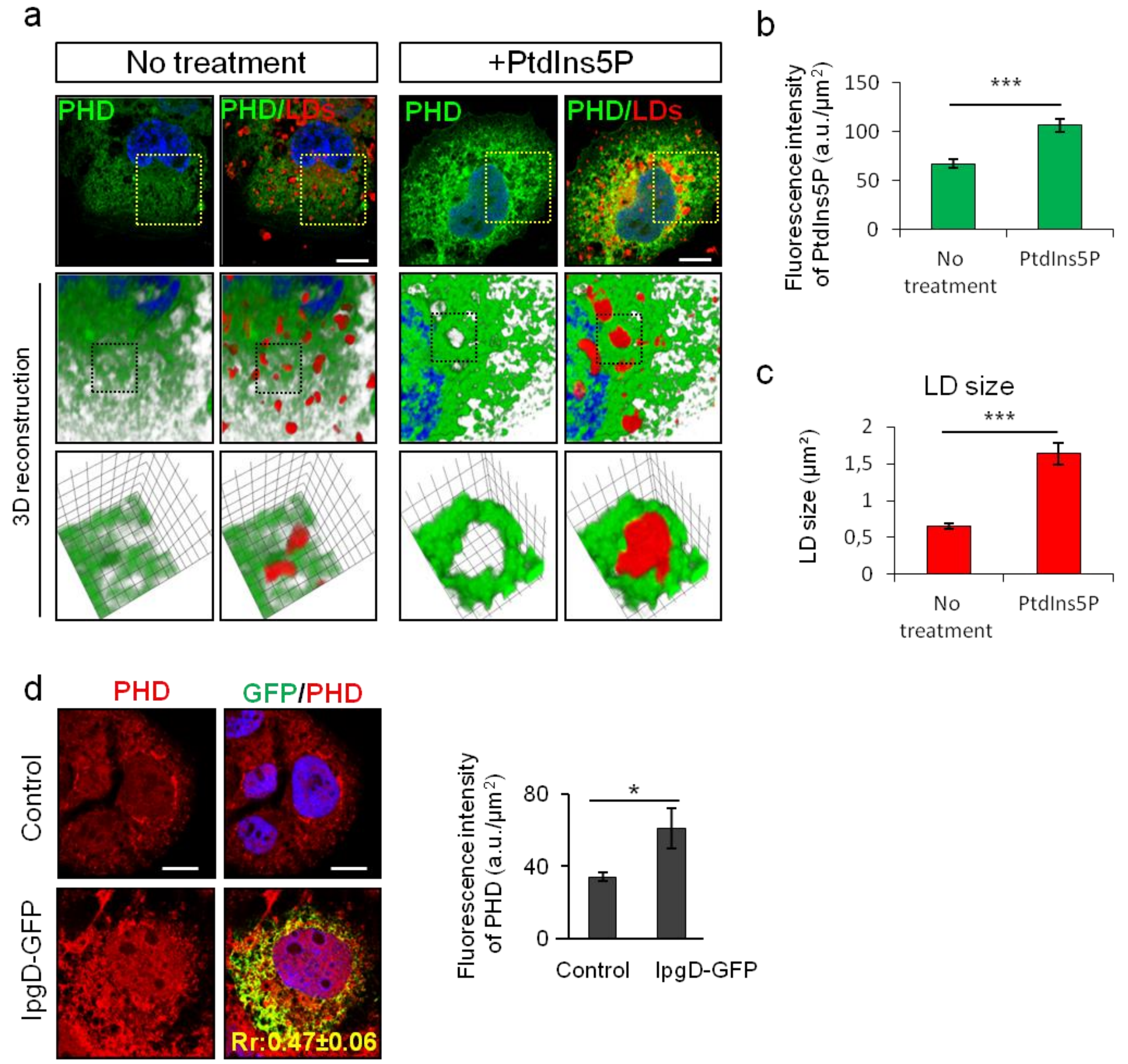

Supplementary Figure 7: PtdIns5P increases by PtdIns5P treatment or IpgD expression

(a) Huh7R cells were untreated or treated with, PtdIns5P at 30 µM for 15 min and stained for PtdIns5P (PHD) (green) and LDs (red). The dot squares indicate the zoom area shown below in higher magnification in 3D reconstruction images. (b) PtdIns5P fluorescence intensity analysis in 60 cells from 3 independent experiments. (c) LD size analysis of 40 cells from 3 independent experiments. (d) Huh7R cells were transfected with IpgD-GFP construct for 48 h and stained for (PHD) (red). Bar graph shows the PtdIns5P fluorescence intensity analysis of 30 cells from 2 independent experiments. Values are means±s.e.m. Student's *t*-test was used. $^*P < 0.05$, $^{***}P < 0.0001$. Scale bar, 10 µm.

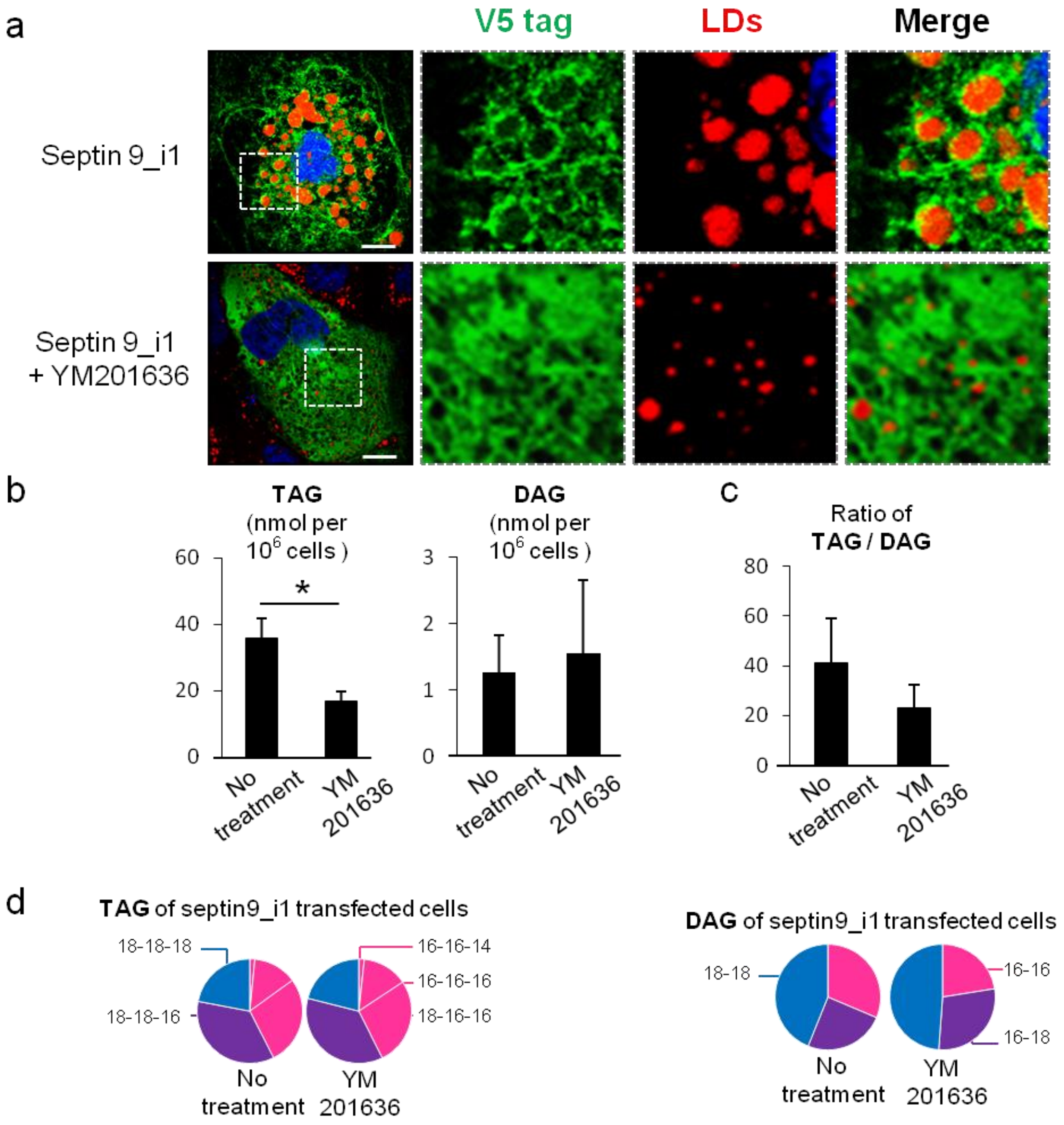

Supplementary Figure 8: YM201636 treatment decreases TAG levels in Huh7R cells

(a) Huh7R cells were transfected with either EV or septin 9_i1 then treated with YM201636 and stained for V5 tag (green) and LDs (red). The dot squares indicate the zoomed area. Scale bar, 10 µm. (b-d) Huh7R cells were transfected with septin 9_i1 then treated or not with YM201636 for analyzing neutral lipids. (b) Bar graph shows triacylglycerol (TAG) and diacylglycerol (DAG) quantities in nmol per 10^6 cells. (c) Ratio of TAG to DAG. (d) Distribution of TAG and DAG fatty acid species according to carbon number in the acyl group. Results were obtained from 3 independents experiments. Values are means±s.e.m. Student's *t*-test was used. *$P < 0.05$.

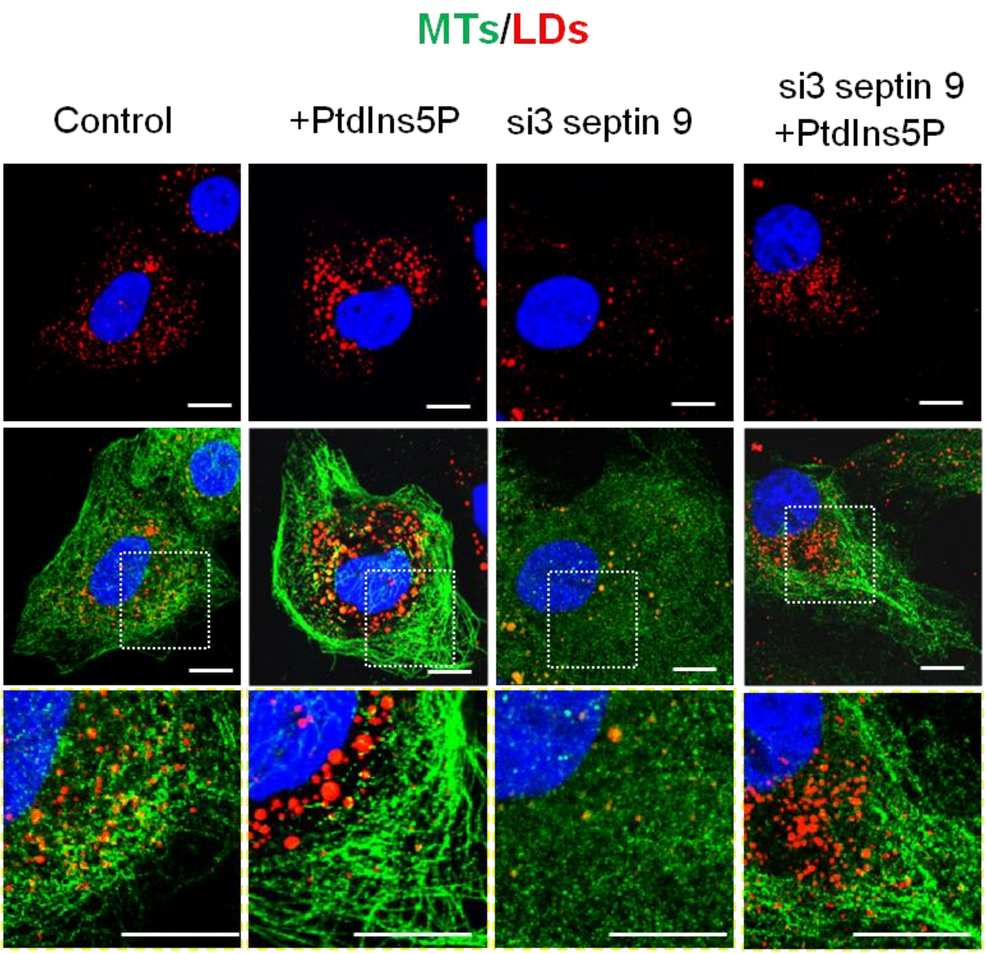

Supplementary Figure 9: PtdIns5P rescues LDs and MTs in septin9 depleted cells

Huh7R cells were transfected with non-targeting or septin 9 siRNA and treated or untreated with PtdIns5P and stained for MTs with β tubulin (green) and LDs (red). The dot squares indicate the zoomed area. Scale bar, 10 µm.

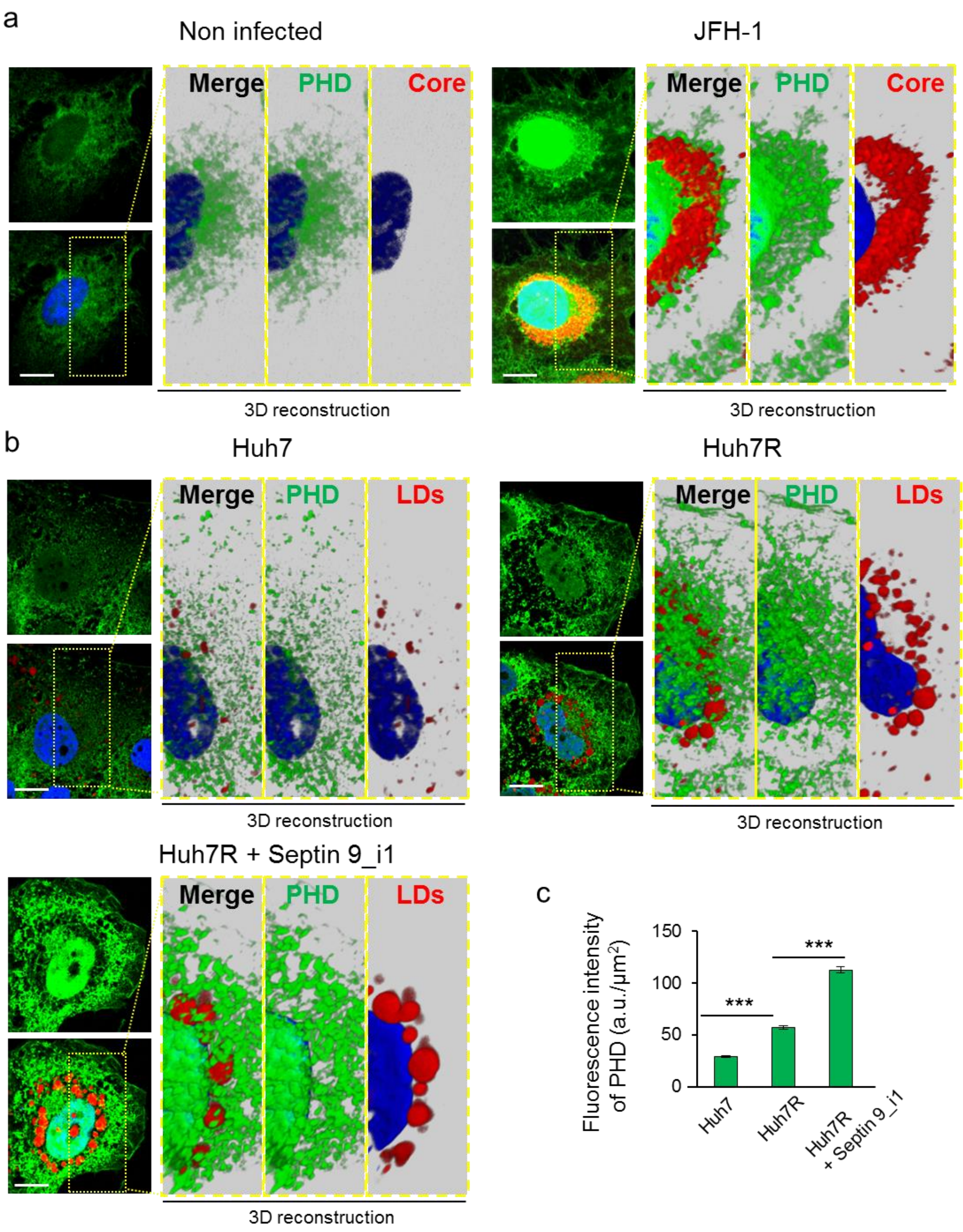

Supplementary Figure 10: HCV and septin 9 expression increase PtdIns5P

(a) Huh7.5 cells were infected or not with JFH-1 for 72 h then fixed and stained for PtdIns5P (PHD) (green) and core (red). The dot yellow rectangles indicate the zoomed area presented at the right in 3D reconstruction images with a gray background. (b) Huh7, Huh7R and Huh7R transfected with septin 9_i1 were fixed and stained for PtdIns5P (PHD) (green) and LDs (red). The dot yellow rectangles indicate the zoomed area presented at the right in 3D reconstruction images with a gray background. (c) PtdIns5P fluorescence intensity measured in 60 cells from 3 independent experiments for each condition. Values are means±s.e.m. Student's *t*-test was used. ***$P < 0.0001$. Scale bar, 10 μm.

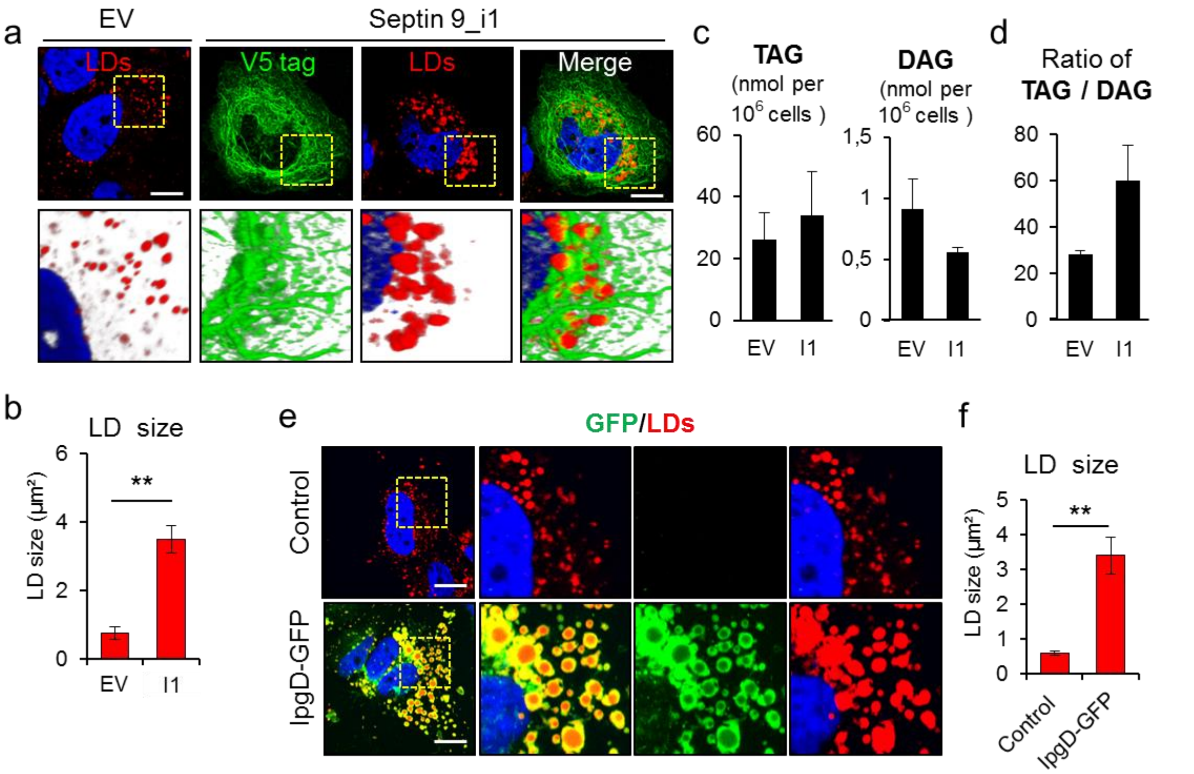

Supplementary Figure 11: Septin9 and IPGD transfection increase LD size in Huh7 cells

(a) Huh7 cells transfected with either empty vector (EV) or septin 9_i1 were stained for LDs (red) and V5 tag (green). Dot yellow squares indicate the zoomed area presented below in 3D reconstruction images. (b) LD size analyzed in 10 cells from 2 independent experiments. (c) TAG and DAG quantities in nmol per 10^6 cells for cells in (a). (d) Ratio of TAG to DAG. (e) Huh7 cells were transfected with IpgD-GFP for 48 h or not (control) and stained for LDs (red). (f) LD size analyzed in 10 cells from 2 independent experiments. Values are means±s.e.m. Student's *t*-test was used. ***P* < 0.001. Scale bar, 10 µm.

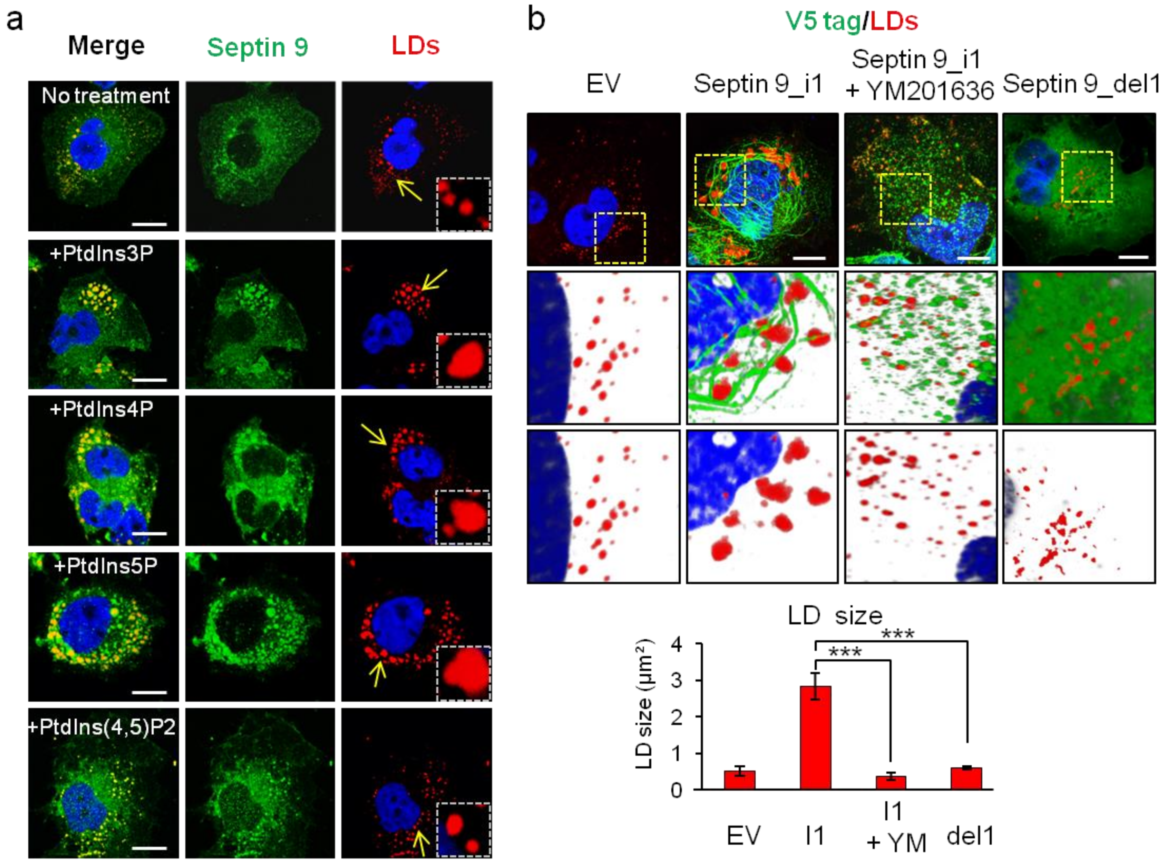

Supplementary Figure 12: Septin9 and PtdIns5P regulate LD size in Huh7 cells

(a) Huh7 cells were treated or not with PtdIns3P, PtdIns4P, PtdIns5P or PtdIns(4, 5)P2 at 30 µM for 15 min and stained for septin 9 (green) and LDs (red). The arrows indicate the zoomed area shown in dot squares. (b) Huh7 cells were transfected respectively with EV, septin 9_i1 treated or not with YM201636 and septin 9_del1 then stained for V5 tag (green) and LDs (red). The dot squares indicate the zoomed area shown to right in a 3D reconstruction image. Bar graph shows LD size in 10 cells from 2 experiments. Values are means±s.e.m. Student's *t*-test was used. ***$P < 0.0001$. Scale bar, 10 µm.

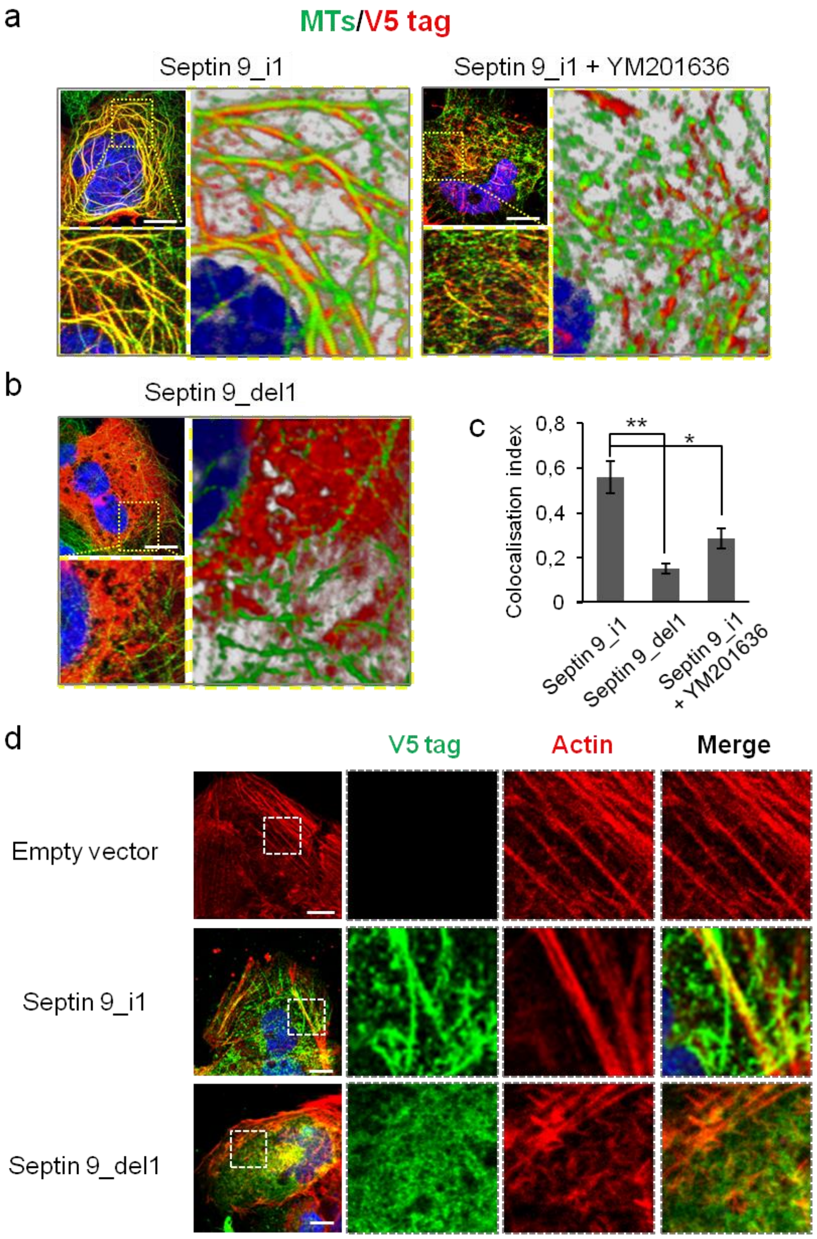

Supplementary Figure 13: YM201636 and PBR deletion affect MTs and actin in Huh7

(a,b) Naïve Huh7 septin 9_i1 transfected cells treated or not with YM201636 (a) and naïve Huh7 cells transfected with septin 9_del1 (b) were stained for V5 tag (red) and microtubules (MTs) with β tubulin (green). The dot squares indicate the zoomed areas shown as a 2D images with a black background, and to the right is a 3D reconstruction image with a gray background. (c) Pearson's correlation coefficient (Rr) measured in 10 cells for each experiment. (d) Naïve Huh7 cells transfected with empty vector, septin 9_i1 and septin 9_del1 constructs were stained for V5 tag (green) and actin (red). The dot squares indicate the zoomed areas shown. Values are means±s.e.m. Student's *t*-test was used. $*P < 0.05$, $**P < 0.001$. Scale bar, 10 μm.

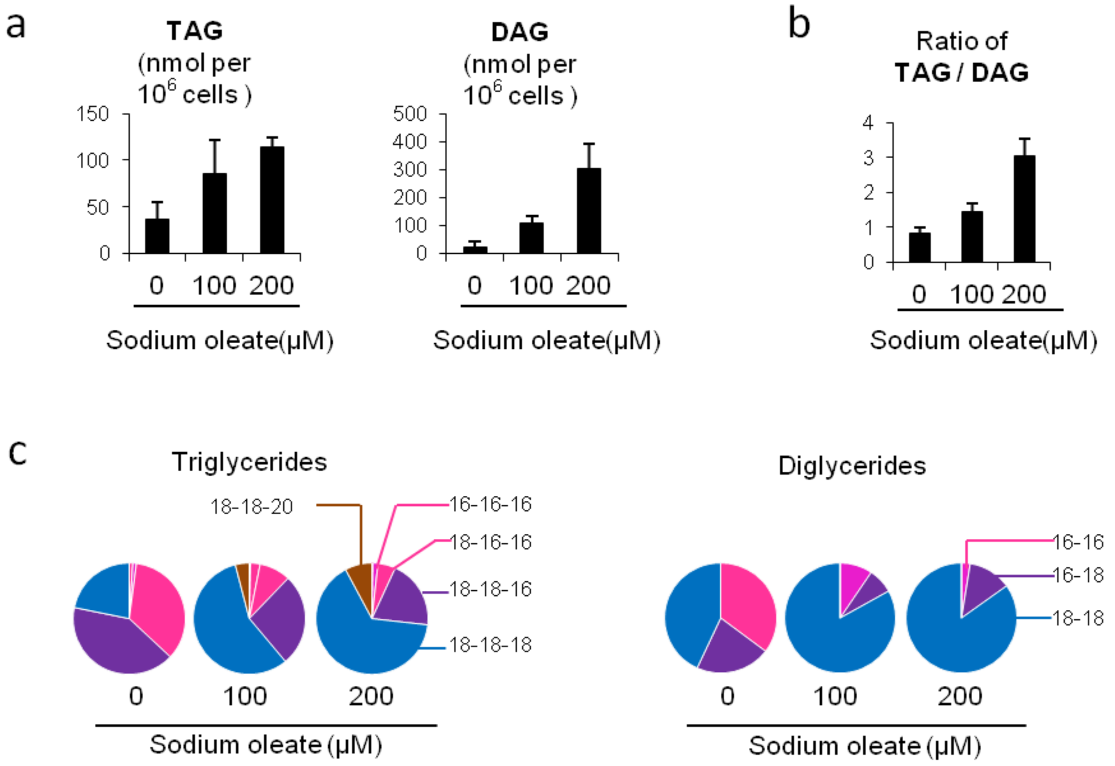

Supplementary Figure 14: Neutral lipids analysis in Huh7 cells treated with oleate

Huh7 cells were incubated with culture media supplemented with sodium oleate-BSA complex at 0, 100, 200 µM for 24 h prior to analysis for neutral lipids. (a) TAG and DAG quantities (nmol per 10^6 cells). (b) Ratio of TAG to DAG. (c) Distribution of TAG, DAG species according to the carbon number in the acyl group. Results obtained from 2 independents experiments.

Figure.2b

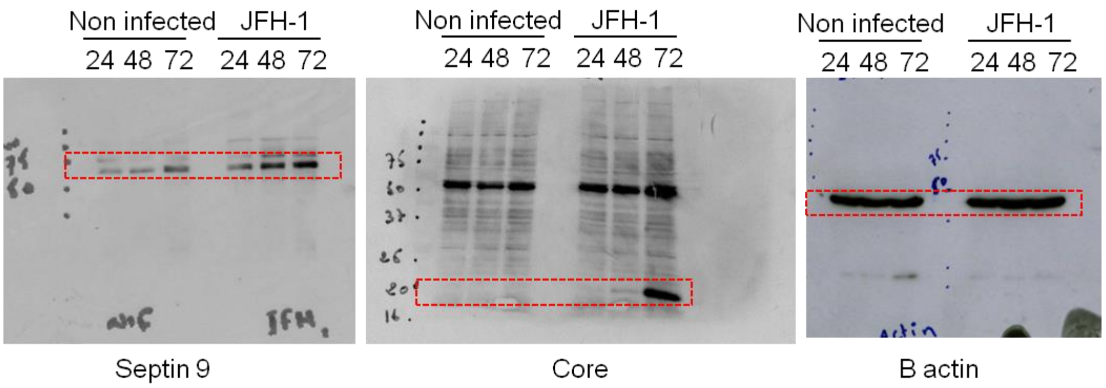

Figure.2c

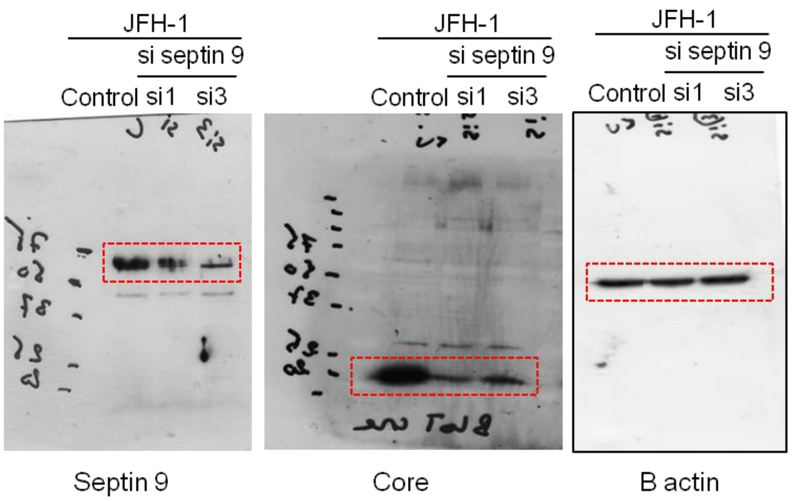

Figure.2g

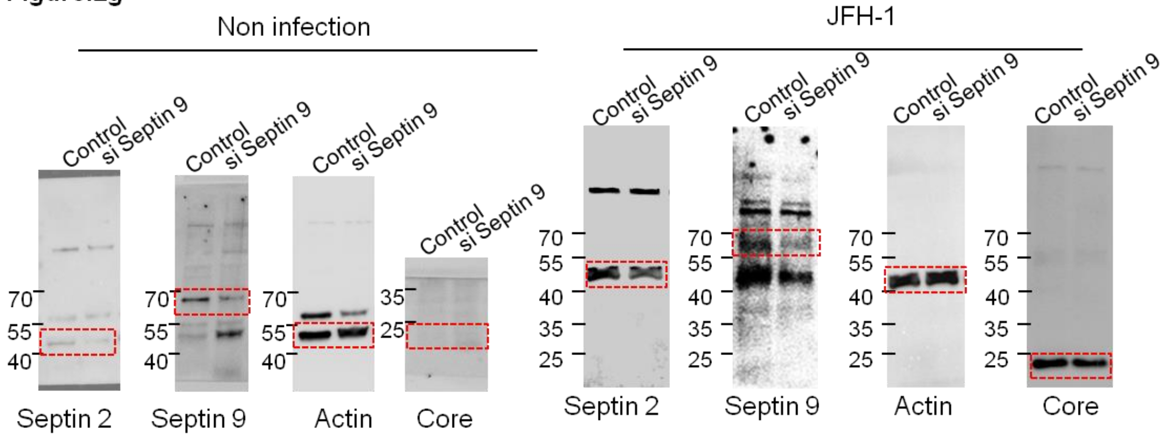

Supplementary Figure 15-1: Uncropped blots

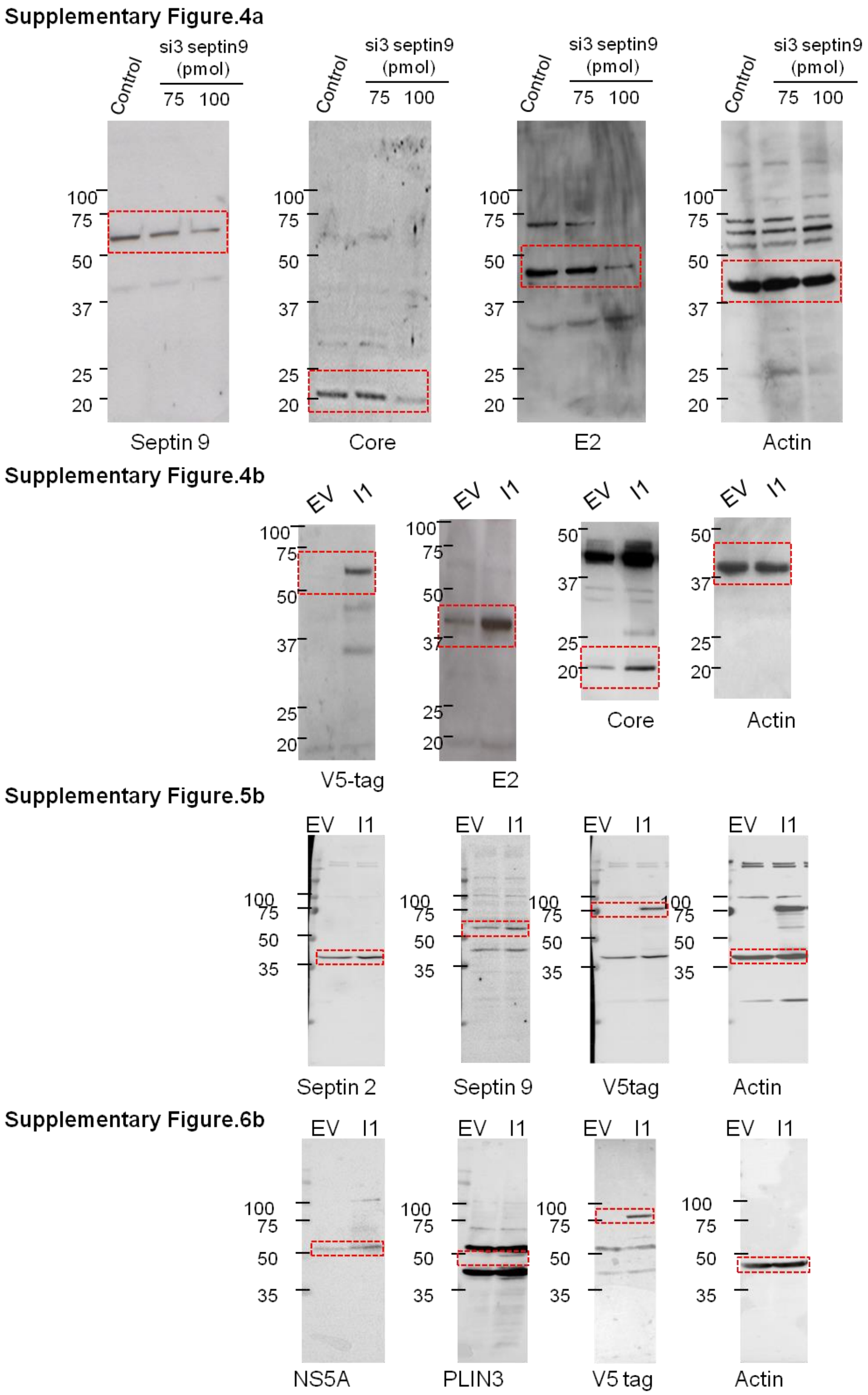

Supplementary Figure 15-2: Uncropped blots

Figure.9b

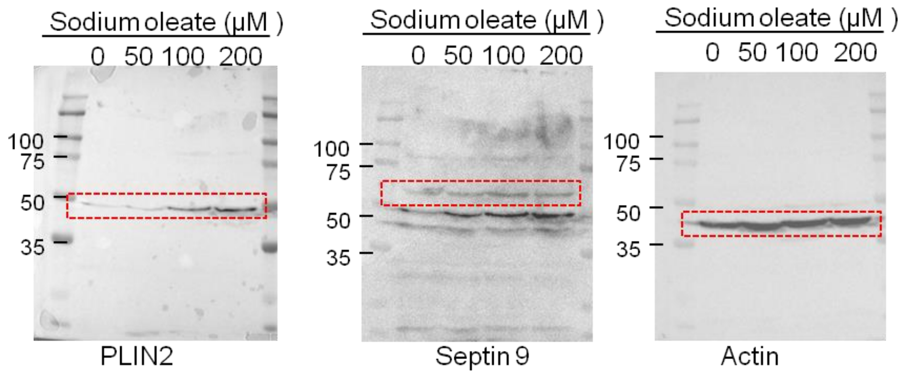

Figure.9c

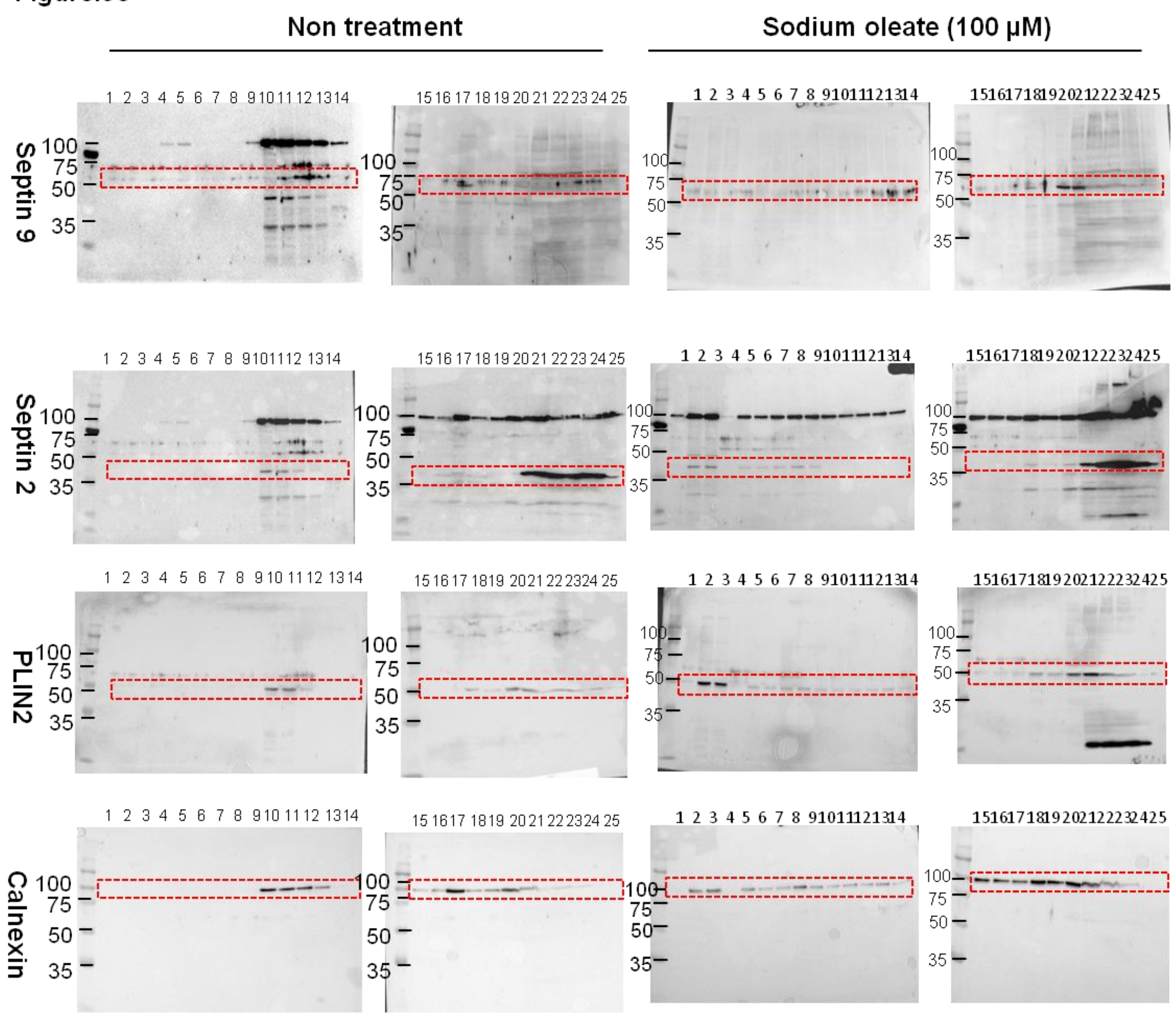

Supplementary Figure 15-3: Uncropped blots

Primer name	Sequences (5'-3')
Mutagenic primer	Forward : CATCCTGGAGCAGATGCAGGGCTTCGAGTTCA
	Reverse : TGAACTCGAAGCCCTGCATCTGCTCCAGGATG
Septin 9 PET21	Forward : CTCCGTCGACAAGCTATGAAGAAGTCTTACTCAGGAGG
	Reverse : GGTGGTGGTGCTCGATCAATGGTGATGGTGATGAT
SEPT 9 for qRT-PCR	Forward : GTCCACTGCTGCCTCTACTTCA
	Reverse : GGACGATGTTGACCACCTTGCT
hG3PDH for qRT-PCR	Forward : AGCCACATCGCTCAGACAC
	Reverse : GCCCAATACGACCAAATCC
HCV	Forward :AGYGTTGGGTYGCGAAAG
	Reverse : CACTCGCAAGCRCCCT
Septin9_ i1	Forward : ATGAAGAAGTCTTACTCAGGAGG
	Reverse : TGGGCCACTGGAGTCACCAAGC
Septin 9 _i2	Forward : ATTCATTCAGCTGAGCCAGG
	Reverse : AGTCCGAAATGATCCCATCC
Septin 9 _i3	Forward : ATGGAGAGGGACCGGATCTCA
	Reverse : CTCGGAGTAGGGGAGTCTGG
Septin 9 _i4	Forward : GCTGGACTCTCTCGCTGACT
	Reverse : CCTGGACAGAGACTGAAGCC
Septin 9 _i5	Forward :AGAGGCCTCCGGAGGAAGAG
	Reverse : CCACCATGATGTTGAACT

Supplementary Table 1: Primer sequences

siRNA	Sequences (5'-3')
si Septin 9 (SEPT9HSS173895) (si1)	Forward : AGGCGUACCGUGUGAAGCGCCUCAA
	Reverse : UUGAGGCGCUUCACACGGUACGCCU
si Septin 9 (SEPT9HSS173897) (si3)	Forward : GCCAUGAAGCAGGGCUUCGAGUUCA
	Reverse : UGAACUCGAAGCCCUGCUUCAUGGC

Supplementary Table 2: siRNA sequences
